# MSstats Version 4.0: Statistical Analyses of Quantitative Mass Spectrometry-Based Proteomic Experiments with Chromatography-Based Quantification at Scale

**DOI:** 10.1021/acs.jproteome.2c00834

**Published:** 2023-04-05

**Authors:** Devon Kohler, Mateusz Staniak, Tsung-Heng Tsai, Ting Huang, Nicholas Shulman, Oliver M. Bernhardt, Brendan X. MacLean, Alexey I. Nesvizhskii, Lukas Reiter, Eduard Sabido, Meena Choi, Olga Vitek

**Affiliations:** 1 Khoury College of Computer Science, Northeastern University, Boston, Massachusetts 02115, United States; 2 University of Wrocław, Wrocław 50-137, Poland; 3 Department of Genome Sciences, University of Washington, Seattle, Washington 98195, United States; 4 Biognosys, Zürich 8952, Switzerland; 5 Department of Pathology and Computational Medicine & Bioinformatics, University of Michigan, Ann Arbor, Michigan 48109, United States; 6 Center for Genomic Regulation, Barcelona Institute of Science and Technology, Barcelona 08003, Spain; 7 Universitat Pompeu Fabra, Barcelona 08002, Spain

**Keywords:** Bioinformatics, Quantitative proteomics, Mass spectrometry, Statistical modeling, Statistical inference

## Abstract

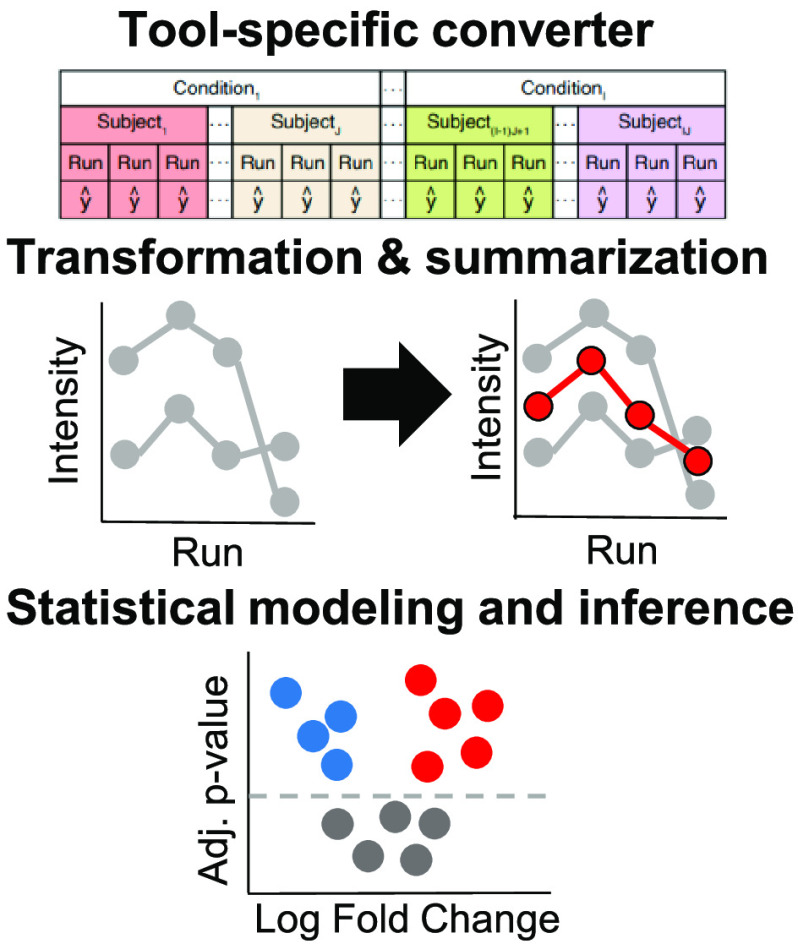

The *MSstats* R-Bioconductor family of packages is widely used for statistical analyses of quantitative bottom-up mass spectrometry-based proteomic experiments to detect differentially abundant proteins. It is applicable to a variety of experimental designs and data acquisition strategies and is compatible with many data processing tools used to identify and quantify spectral features. In the face of ever-increasing complexities of experiments and data processing strategies, the core package of the family, with the same name *MSstats*, has undergone a series of substantial updates. Its new version *MSstats* v4.0 improves the usability, versatility, and accuracy of statistical methodology, and the usage of computational resources. New converters integrate the output of upstream processing tools directly with *MSstats*, requiring less manual work by the user. The package’s statistical models have been updated to a more robust workflow. Finally, *MSstats*’ code has been substantially refactored to improve memory use and computation speed. Here we detail these updates, highlighting methodological differences between the new and old versions. An empirical comparison of *MSstats* v4.0 to its previous implementations, as well as to the packages *MSqRob* and *DEqMS*, on controlled mixtures and biological experiments demonstrated a stronger performance and better usability of *MSstats* v4.0 as compared to existing methods.

## Introduction

Technological advances in bottom up liquid chromatography coupled with high-resolution tandem mass spectrometry (LC-MS/MS) have facilitated the quantification of changes in protein abundance across many conditions.^1^ The experiments have increasingly complex experimental designs differing in number and type of conditions (group comparisons or repeated measures), and number and type of replicates (biological replicates, technical replicates, fractionation).^2,3^ Additionally, numerous versions of chromatography-based acquisition methods, such as Data-Dependent Acquisition (DDA), Selected Reaction Monitoring (SRM), and Data-Independent Acquisition (DIA), now offer effective and complementary strategies for relative protein quantification with high accuracy and high throughput.^4^ The experiments generate mass spectra, with features corresponding to peptide ions for DDA, peptide transitions for SRM, and peptide ions and fragment ions for DIA.

Regardless of the experimental aims and design, software tools are required to identify and quantify proteins in the resulting MS spectra. A variety of computational spectral processing tools, such as MaxQuant,^5^ Skyline,^6^ and Spectronaut,^7^ extract, identify, and quantify features from the acquired spectra.^8^ These tools are developed by different groups, optimized for different instruments and workflows, and make different decisions when feature identification or quantification are challenging. For example, the tools differ in strategies for quantifying feature intensities in individual LC-MS runs, e.g., peak area or peak height at apex. They also differ in reporting the intensity of the monoisotopic peak, the sum of multiple isotopic peaks, or relying on more advanced procedures. Moreover, the tools make different choices for reporting outlying or missing values, which occur in the presence of truncated or overlapped peaks, or when the abundance of an analyte is below the limit of detection.

The complexities of the experimental designs, data acquisitions and data processing create major challenges for the downstream statistical analyses, and may lead to inconsistent or erroneous results.^9^ To accommodate these new challenges, methods for statistical analyses and their implementations must grow in parallel. Statistical methods must be both versatile enough to reflect various scientific questions and experimental designs, and robust enough to not overfit to a particular experiment, experimental design, or data processing tool. Implementations must utilize memory-efficient and time-efficient data structures and algorithms that scale to large data sets.^10^

A variety of statistical methods for detecting differentially abundant proteins have been developed to address these challenges. We loosely classify the existing methods into two groups: two-step and feature-based. Two-step methods first summarize the intensities of all the features of a protein in a run, and then subject the summaries to statistical modeling. In contrast, feature-based methods take as input quantified features, and specify a full statistical model at the feature level directly.^11,12^ For example, *MSqRob*(13,14) includes functionalities for both a feature-based workflow, as well as summarization functionality for a two-step workflow. The package utilizes a linear mixed effects model to detect differential proteins and includes functionality to apply empirical Bayes variance estimation.^15^ Meanwhile, *DEqMS*(16) takes as input protein-level summaries, reported by a data processing tool such as MaxQuant, and leverages the methods in the Limma R package^17−19^ to moderate protein variance’s through grouping proteins by their number of features in order to identify differentially abundant proteins. Other packages of note include *proDA*,^20^ a two-step method which focuses on missing data and implements a probabilistic dropout based model to combine missing and observed values, *pmartR*,^21^ another two-step method which focuses on the quality control, preprocessing of MS data, and Analysis of Variance(ANOVA)^22^ for statistical analysis, and *DAPAR*,^23^ which implements a two-step method in a graphical user interface. Other packages, such as *QFeatures*(24) and *msImpute*,^25^ focus on the first step of the two-step workflow, implementing functionalities for missing value imputation, and data preprocessing.

Although software tools implementing many methods exist, their functionalities often have limitations. First, the methods do not easily integrate with upstream spectral processing tools. The outputs of these tools typically need to be manually converted into the correct format before it can be used for the statistical analyses. Only a subset of implementations include functionality for data processing such as feature filtering, normalization, and missing value imputation, leaving the user to rely on other resources such as the *QFeatures*, or implement the steps on their own. The latter solution is particularly undesirable, as custom data processing leads to irreproducible results. Beyond data processing, while all the workflows provide functionalities for group comparison, their ability to distinguish biological and technical replicates is not always clear. This is undesirable for complex experimental designs, such as time-course and paired designs, where each replicate is measured in multiple conditions. Additionally, some of the methods require users to manually specify the model that fits their data. These methodological aspects challenge users with no statistical background, and can result in incorrectly specified models. Finally, not every implementation is optimized in terms of runtime and computational memory requirement for large data sets.

Here, we introduce *MSstats* version 4.0 (v4.0), a statistical methodology and core package in the family of R/Bioconductor packages designed for statistical analysis of experiments with chromatography-based quantification. Compared to the previous versions of *MSstats*, *MSstats* v4.0 includes a series of methodological and technical improvements. Specifically, compared to *MSstats* v2.0, *MSstats* v3.0 includes substantial methodological improvements. These include updates to upstream data processing, such as missing value imputation, and switching from a feature-based to two-step modeling method. *MSstats* v4.0 implements the same statistical models as *MSstats* v3.0, but with a substantially refactored code that improves both the processing speed and memory use. While *MSstats* has undergone many methodological and technical improvements in recent years, there has not been a comprehensive review of the package and methods since *MSstats* v2.0.

Additionally, since its original publication, *MSstats* has expanded into a broad family of R/Bioconductor packages and methods. *MSstats* v4.0 is now designed for label-free acquisition and detection of relative changes in protein abundance, while other packages in the family address different types of experimental designs and biological objectives. Specifically, *MSstatsTMT*(26) is designed for experiments acquired via tandem mass tag (TMT) labeling. *MSstatsPTM*(27) focuses on experiments studying post-translational modifications (PTMs). Finally, *MSstatsShiny*(28) is an R-shiny based graphical user interface (GUI), which makes the *MSstats* methods accessible to users without programming background. While *MSstats* v4.0 itself is not directly designed for these specific use-cases, it serves as a backend for many. *MSstatsTMT* directly applies the summarization methods in *MSstats* v4.0 to each TMT mixture. *MSstatsPTM* utilizes the models in *MSstats* v4.0, while adding a statistical correction to remove confounding with the unmodified protein. *MSstats* v4.0 is at the core of all packages in the *MSstats* package family and the methods are leveraged without any input required by the user.

*MSstats* v4.0 includes direct converters for popular data processing tools. Converters for label-free DDA experiments include MaxQuant,^5^ Fragpipe,^29^ Skyline,^6^ OpenMS,^30^ Proteome Discoverer (Thermo Scientific), and Progenesis QI (Nonlinear Dynamics/Waters). SRM spectra can be quantified by Skyline, and MultiQuant (Sciex). DIA spectra can be processed by Skyline, Spectronaut^7^ (Biognosys), DIA-Umpire,^31^ OpenSWATH,^32^ and DIA-NN.^33^ After conversion, *MSstats* v4.0 includes multiple options for upstream data processing, including feature filtering and missing value imputation, providing users flexibility in preparing their data for modeling. *MSstats* v4.0 implements a two-step modeling method, first summarizing the feature intensities, and then fitting a linear mixed effects model to the summarized data. *MSstats* automatically adjusts the linear mixed effects model to fit the specific experimental design, greatly easing implementation complexity. Finally, *MSstats* v4.0 leverages several computational strategies to improve memory use and computation speed, including moving high-resource functions to the C++ programming language, and implementing a new workflow for larger-than-memory input files.

Here we empirically compared *MSstats* v4.0/v3.0 to *MSstats* v2.0 to *MSqRob*, and *DEqMS* on three controlled mixtures and two biological experiments. We demonstrated that the two-step statistical methodology is more versatile, being applicable to time-course and paired designs, as well as more accurate compared to the models in *MSstats* v2.0. *MSstats* v4.0 outperformed existing methods, while being easier to implement by users with limited statistical background. Finally, we contrasted the computational resource usage between *MSstats* v2.0, v3.0, and v4.0 to highlight recent technological improvements. The package is open source and is available on Bioconductor and Github.

## Experimental Procedures

### Data Overview and Availability

Table 1 summarizes the data sets included in this manuscript. Three controlled mixtures were obtained by spiking proteins in known concentrations into a complex background or by diluting the same parent mixture. They covered a broad range of known fold changes between conditions and represent two DDA and one DIA data acquisition strategy. Two biological investigations demonstrated the applicability of the proposed approach across different experimental designs and acquisition strategies. The true composition of the biological experiments were unknown and the statistical methods could only be evaluated in terms of the differences between the outputs of *MSqRob*, *DEqMS*, *MSstats* v2.0, and *MSstats* v4.0. Finally, two simulated out of memory data sets were generated to illustrate the functionalities of *MSstatsBig*.

**Table 1:** Experimental Data Sets in this Manuscript^a^

	Experimental Design				Data Availability
Data Set	Type	No. of Conditions	No. of Bio. Replicates	No. of Tech. Replicates	(MassIVE.quant or R Package)
1: Controlled Mixture–DDA–MaxQuant^9^	Group	4	1	3	RMSV000000249.2
2: Controlled Mixture–DIA–Spectronaut^34^	Group	2	1	3	RMSV000000250.2
3: Controlled Mixture–DDA–Skyline^35^	Group	5	1	3	RMSV000000261.1
4: Mouse–DDA–MaxQuant^36^	Paired	2	6	2	RMSV000000292.1
5: *S. cerevisiae*–DIA–Skyline^37^	Time course	6	3	1	RMSV000000251.1

a “Data Set” is the code name of the data set. “Type” is the type of experimental design (group comparison, paired, or time course. “No. of Conditions” states the number of conditions in the data set. “No. of Bio. Replicates” states the number of biological replicates per condition. “No. of Tech. Replicates” states the number of technical replicates per biological replicate. More details about experimental design and data processing steps for each data sets are available in next section, as listed in “Details in” column. The details of the experimental design, the raw data, the reports, the analysis scripts, the intermediate data processing output files including quantification, testing results are available in MassIVE.quant.

Details of data processing, software versions, R scripts with *MSstats* analyses, and result of statistical analyses for all data sets are available in MassIVE.quant (with direct links available in Table 1).

### Data Set 1: Controlled Mixture–DDA–MaxQuant^9^

The experiment was conducted as part of the 2015 study of the Proteome Informatics Research Group (iPRG) of the Association of the Biomedical Resource Facilities (ABRF). Six proteins were spiked into samples with *S. cerevisiae* proteome as the background. Four proteins were spiked with four different concentrations, forming a Latin Square design. Two more proteins were spiked in another four different concentrations in the same four biological samples. The concentrations are summarized in Table 2. Data from four mixtures were acquired in DDA mode with triplicate MS runs, resulting in 12 MS runs in total. Raw data were processed with MaxQuant.

**Table 2:**
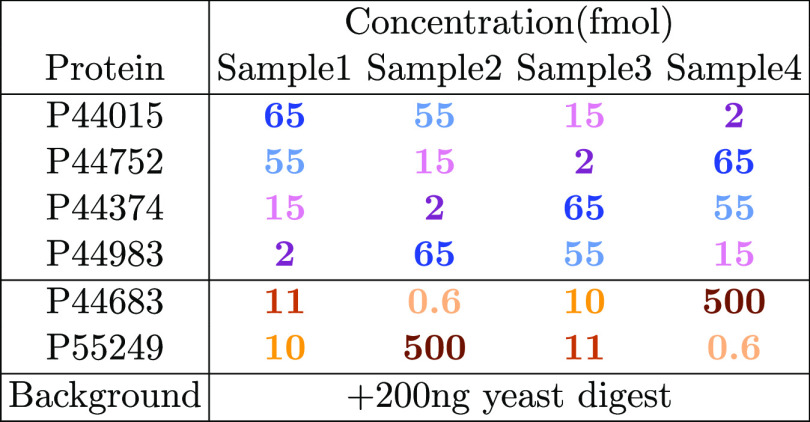
DDA:Choi207 Protein IDs and Concentrations of Spike-in Proteins

### Data Set 2: Controlled Mixture–DIA–Spectronaut^34^

This study prepared two hybrid proteome samples, A and B, consisting of tryptic digests of human, *S. cerevisiae*, and *E. coli* proteomes. The proteomes were mixed in defined proportions, to yield expected peptide and protein ratios (A/B) of 1:1 for human, 2:1 for *S. cerevisiae* and 1:4 for *E. coli* proteins. Data from two mixtures were acquired in DIA mode with triplicate MS runs, resulting in 6 MS runs, and processed with Spectronaut. This manuscript used the raw files acquired on TripleTOF 6600, Iteration 2, with SWATH window number 64.

### Data Set 3: Controlled Mixture–DDA–Skyline^35^

Thirty proteins were prepared at 1.5 pmol/μL in three different subsets of 10 proteins each (Table 3). Proteins from these subsets were spiked into a 15 μg *E. coli* background in different amounts of five mixtures. The final amount of each protein in each mixture was either 100, 200, or 400 fmol/μg of *E. coli* background, indicated as 1, 2, and 4 in Table 4. Data from the mixtures were acquired in DDA mode with triplicate MS runs, resulting in 15 MS runs in total. Raw data were processed with Skyline.

**Table 3:** SwissProt Accession Numbers of Proteins Spiked into the *E. coli* Background in Data Set 3: Controlled Mixture–DDA–Skyline

Subset 1		Subset 2		Subset 3	
Accession Number	Molecular Weight	Accession Number	Molecular Weight	Accession Number	Molecular Weight
P02701	16	P00915	29	Q3SX14	81
P00711	16	P02787	77	P00563	43
Q29443	78	P02663	24	P02769	69
Q29550	62	P01008	53	Q58D62	48
P0CG53	8	P00921	29	P00698	15
P68082	17	P05307	57	P00004	12
P00432	60	P61769	14	P00711	14
P02754	20	P02662	24	P00442	33
P24627	78	P01012	45	P01133	134
P80025	81	P02666	25	P02753	23

**Table 4:**
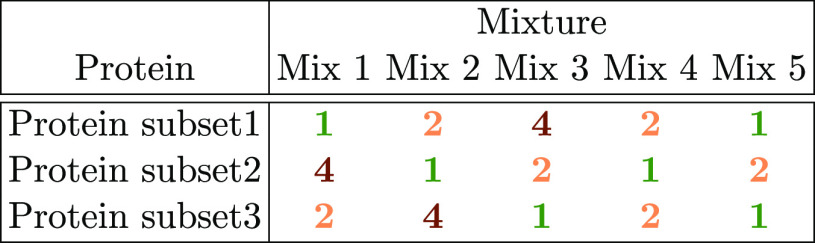
Data Set 3: Controlled Mixture–DDA–Skyline Relative Protein Concentrations

### Data Set 4: Mouse–DDA–MaxQuant^36^

This experiment investigated Ataxin-2 (ATXN2) deficiency through Atxn2-knockout (Atxn2-KO) mice. Liver samples were taken from six wild type (WT) and six Atxn2-KO mice in a balanced paired design. Data were acquired in DDA mode with 1 technical replicate and processed with MaxQuant. Details on the specific instrument and instrument settings are available in the original publication.^36^ We evaluated the ability of the statistical methods to correctly estimate the fold change and identify differentially abundant proteins between conditions.

### Data Set 5: *S. cerevisiae*–DIA–Skyline^37^

Cells of *S. cerevisiae* were cultured in biological triplicates, and sampled at six time points (0 min (T0), 15 min(T1), 30 min (T2), 60 min (T3), 90 min (T4), 120 min (T5)) after osmotic stress. Data were acquired in SWATH/DIA mode, and processed using Skyline. The analysis used additional information from 8 technical replicate MS runs on the same samples, and limited the analyses to peptides that were detected in at least 4 runs and CV less than 20% in these 8 runs. In this manuscript we compared five time points relative to T0 (T1 vs T0, T2 vs T0, T3 vs T0, T4 vs T0, T5 vs T0). The true changes in protein abundance were unknown. We evaluated the ability of the statistical methods to identify differentially abundant proteins between conditions.

### Simulated Out-of-Memory Data

To illustrate the performance of *MSstatsBig*, we generated two out-of-memory data sets based on Data set 2: Controlled Mixture–DIA–Spectronaut. The first data set was created by replicating the Controlled Mixture 15 times, and the second was created by by replicating it 30 times. The 15 copy data set was 17.3 GB in size, while the 30 copy was 34.6 GB.

## Background on Existing Statistical Methods for Differential Analysis of MS-Based Proteomics

### Statistical Methods with Bioconductor Implementation

**Figure 1:**
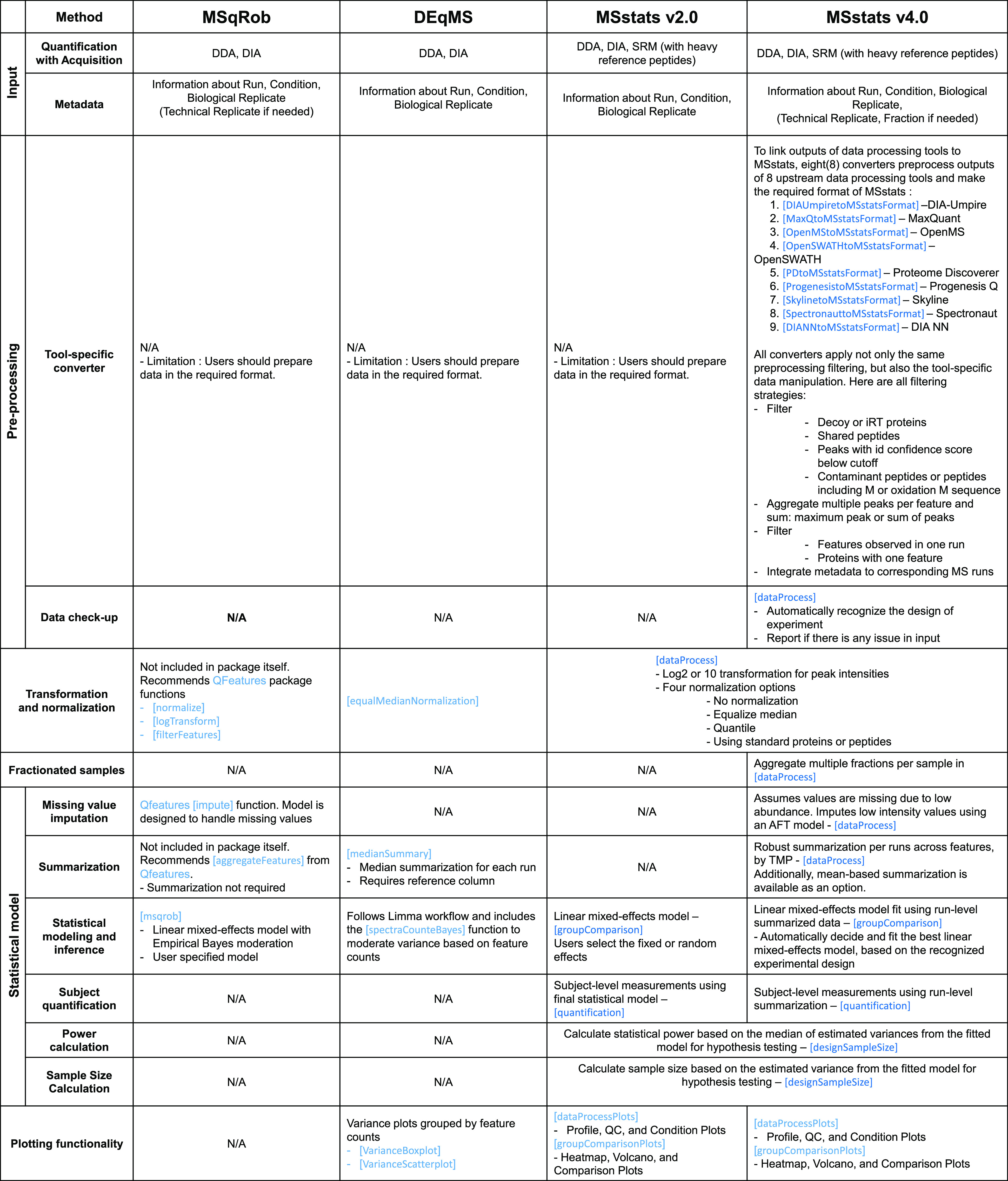
Functionality and workflow comparison between *MSstats* v4.0, *MSstats* v2.0, *MSqRob*, and *DEqMS*. function [function] indicates the corresponding function name in *MSstats*, *MSqRob*, or *DEqMS*. N/A indicates functionality that was not available.

In this section we provide additional details on *MSqRob* (implemented in the *msqrob*2 package on Bioconductor) and *DEqMS*. These two packages are closest to the scope of *MSstats* in that they (1) are designed for quantitative proteomic experiments with chromatography-based quantification (i.e., DDA, DIA or SRM data acquisitions), (2) have open-source implementations, (3) are compatible with multiple data processing tools, and (4) are easily installed and used via Bioconductor. Figure 1 overviews the functionalities of these packages, and additional details are given below.

#### MSqRob

The *MSqRob*(13,14) analysis workflow is documented in the package vignette. The workflow takes as input manually converted feature level data in a custom format. Once in the required format, the data is input into the *readQFeatures*() function from the *QFeatures*(24) packages. The vignette suggests multiple preprocessing steps using both functions from the *QFeatures* package and manual implementations. The package includes methods for dealing with missing values, both converting censored values (such as 0) to NA, as well as missing value imputation. The package vignette itself does not mention missing value imputation, however the *impute*() function from *QFeatures* can be used to perform one of 13 imputation methods. Finally, *MSqRob* utilizes the *normalize*() function from *QFeatures* for normalization, which gives users a wide variety of options to choose from.

After data preprocessing, *MSqRob* includes summarization functionality (via *QFeatures*). The package does not require summarization, and provides the option for differential analysis using the individual protein features or summarized intensities. Multiple summarization strategies are available, including Tukey’s median polish,^38^ robust regression summarization, or simply taking the mean, median, or sum of the protein features. Finally, the package performs differential analysis using a linear mixed effects model (*msqrob*() function). The users must specify the correct model for their data, and this requires potentially nontrivial statistical expertise. Multiple options exist for estimating the parameters of the models, including ridge penalty, M-estimation, and Empirical Bayes. Once the model is fit, contrast-based analysis is used for group comparison. Finally, the package itself does not include plotting functionalities, although the vignette leverages plotting functions from the *Limma* package for manually created plots.

#### DEqMS

The *DEqMS*(16) analysis workflow is described in the package vignette. The package provides functionality for both label-free and tandem mass tag (TMT) labeled workflows. The package does not include any direct converters for spectral processing tools and requires manual conversion by the user. For data preprocessing, the package includes a normalization function (*equalMedianNormalization*()) which implements median normalization. Beyond normalization, there are no other data preprocessing options implemented in the package, although the vignette does suggest a few manual prepossessing steps. For summarization, the package includes the summarization function *medianSummary*(). The summarization function requires the user to select a reference column, which is used to calculate the relative peptide ratios. In the function example, this reference column is a reference channel in a TMT experiment. There was no example of using this function for label-free experiments.

For modeling, *DEqMS* implements a version of *Limma* which leverages feature counts for variance moderation. The model takes as input run-level summaries for each protein, grouped into an individual column per MS run. The model is implemented as a 4 step workflow. The first 3 steps follow the classic *Limma* modeling workflow. This includes fitting a linear model, contrast based group comparison, and variance moderation. After the *Limma* model is fit, *DEqMS* groups proteins with the same number of features together, and individually moderates the variance of the feature-groupings together. In this way, proteins with low features do not have their variances artificially reduced by proteins with high features, and vice versa. *DEqMS* includes two plotting functions (*VarianceBoxplot*() and *VarianceScatterplot*()) to visualize the results of the feature grouping variance moderation. Other plots of the results must be manually created.

### Previously Published Version of MSstats

Figure 1 details the implementation of *MSstats* v2.0. *MSstats* v2.0 supported DDA, DIA, and SRM acquisitions, however it did not support more complex designs, including those with technical replicates, fractionation, time course, and paired designs. Similarly to other existing methods, *MSstats* v2.0 did not include any tool-specific converters. The users had to manually convert their data set into the required format, which was a challenge for users without an in depth knowledge of the *MSstats* formatting and advanced computational skills. In terms of data preprocessing, *MSstats* v2.0 included limited options and functionalities. The *dataProcess*() function gave users the option to either log_2_ or log_10_ transform peak intensities, and four options for data normalization (equalizing medians across runs, quantile normalization, peptide or protein standard normalization, or not performing normalization). There were no options for missing value imputation, feature filtering, or feature summarization.

*MSstats* v2.0s *groupComparison*() function implemented a linear mixed-effects model for differential analysis. The model was feature-based, taking as input quantified features, and specifying a full statistical model at the feature level. This resulted in high computational complexity and issues of numeric stability of the model fit in some cases.Additionally, while *MSstats* v2.0 implemented a mixed-effects model, it required the user to manually specify the fixed and random effects. This was a challenging step for users with limited statistical background. Finally, after modeling was performed, *MSstats* v2.0 implemented functionality for future experiment planning, including power and sample size calculations in the *designSampleSize*() function.

## Results

### Statistical Methods and Implementations in MSstats v4.0

#### Adaptive Converters Statistically Interpret and Format the Outputs of Data Processing Tools

**Figure 2:**
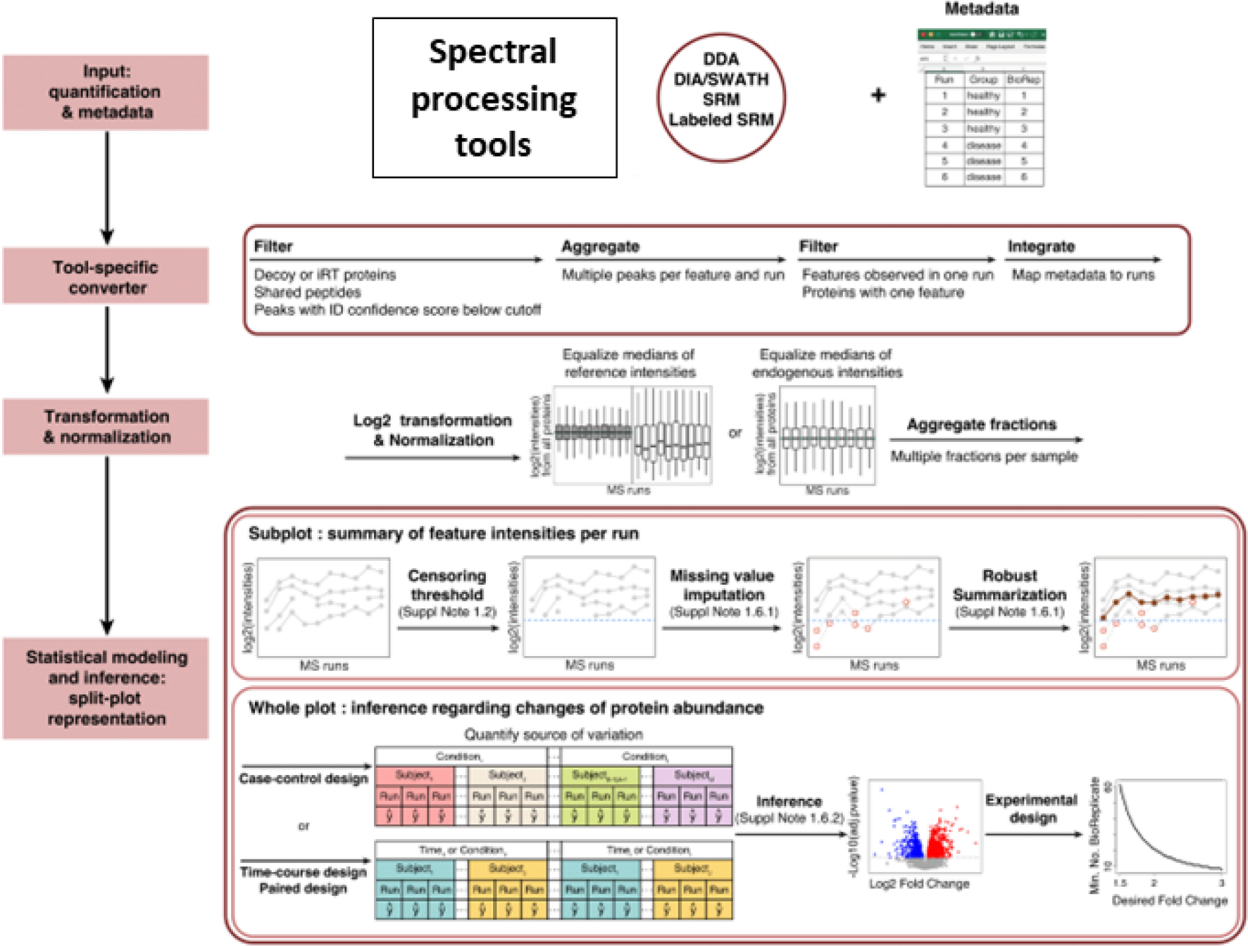
*MSstats* v4.0 workflow and place in the bottom up LC-MS/MS proteomics analysis pipeline *MSstats* takes as input the output of spectral processing tools used for identification and quantification and an annotation file matching MS runs to experimental metadata, such as conditions and biological replicates. Tool-specific converters perform data filtering and aggregation. Next the data is transformed onto the log_2_ scale and normalized. Finally, statistical modeling and inference is performed, with optional plotting to view the results.

*MSstats* v4.0 takes as input the log_2_-intensities of peaks obtained by data processing tools, step 1 of Figure 2, (as opposed to ratios of peak intensities between the groups), to facilitate statistical modeling.^39^ Since spectral processing tools differ in both analytical strategies and output formats, *MSstats* v4.0 employs tool-specific converters (Step 2 of Figure 2), which ensure that the output of the tools is interpreted and formatted correctly.

Converters in *MSstats* v4.0 follow a consistent workflow implemented in the Bioconductor package *MSstatsConvert*.^40^ First, raw data from the tools are reshaped into long format, with columns denoting protein ID, peptide sequence, precursor charge, fragment ion and product charge, information about labeling, run ID, and intensity value. Each run is then annotated with labels for condition and biological replicate using the information provided in the accompanying “annotation” file. The “annotation” file maps run names to the experimental design, and must be created manually by the user. Details on how to create this file and example “annotation” files for different experimental designs can be found in Supporting Information Section 1. This minimum set of columns constitutes the *MSstats* format. Whenever applicable, the user can add information about the ID of technical run and fraction, along with optional columns for use with filtering. This step of the workflow is tool-specific.

The following steps are generic and can be applied to any data set in the described format. They consist of filtering (for example by Q-values or removing contaminants), handling isotopic peaks, removing features with few measurements across runs, removing shared peptides (i.e., peptides assigned to more than one protein by the spectral processing tool), aggregating features with duplicated measurements in a run, optional filtering of proteins identified by a single feature, and adding annotation. These operations are implemented by the *MSstatsPreprocess*() function in *MSstatsConvert* package. The next step of the workflow selects or aggregates fractionated runs and creates balanced design, with one row in the data table for every run for each feature, even if the intensity value is missing. These operations are performed by the *MSstatsBalancedDesign*() function from *MSstatsConvert* package.

The *MSstatsConvert* package enables creation of new converters consistent with the intended *MSstats* workflow. With this package, users can create converters for yet unsupported tools (including custom workflows) using the same logic as in *MSstats* converters with minimal coding, as the only tool-specific step is the reshaping of raw data.

**Figure 3:**
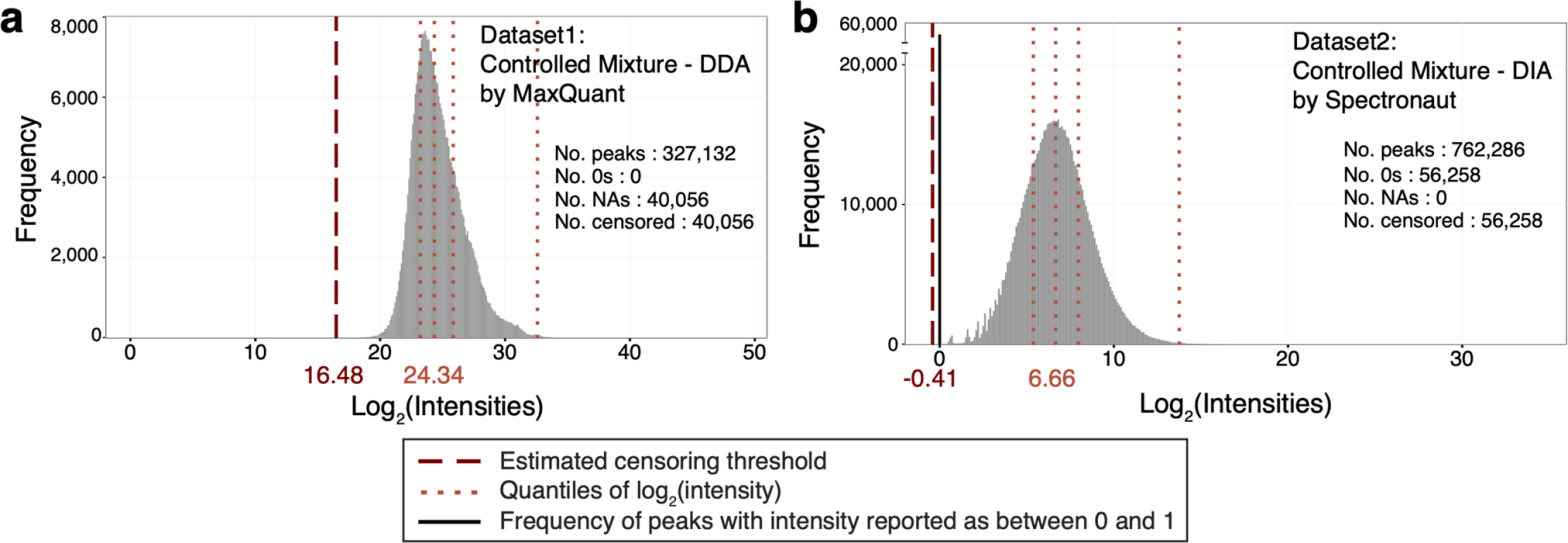
Distribution of log_2_ transformed and normalized intensities, after normalization. (a) Data set 1: Controlled Mixture–DDA–MaxQuant. (b) Data set 2: Controlled Mixture–DIA–Spectronaut. Median normalization equalized the medians of log_2_-intensities of all the features between the MS runs. “No. peaks” reports the number of peaks with log_2_ intensity >0. “No. 0s” is the number of log_2_ intensity = 0, “No. NAs” is the number of intensities reported as “NA” by the data processing tool. “No. censored” is the number of censored intensities as defined by *MSstats* v4.0. Dotted orange lines indicate the 25, 50, 75, and 99.9th percentiles (*q̂*_25_, *q̂*_50_, *q̂*_75_, and *q̂*_99.9_) of the log_2_ intensities exceeding 0. Dashed dark red lines indicate the censoring threshold, estimated by *MSstats* v4.0.

Figure 3 visualizes feature intensities from two data sets in the Experimental Procedures section: Data Set 1: Controlled Mixture–DDA–MaxQuant and Data Set 2: Controlled Mixture–DIA–Spectronaut. Since spectral processing tools differ in their approaches to reporting missing values, the number of 0 and “NA” values varied across tools. Data set 1 quantified by MaxQuant reported no small intensities and no 0s, but a large amount of “NA” values. Meanwhile Data set 2 quantified by Spectronaut reported a large amount of 0s but no “NA” values. MaxQuant reports missing values as “NA”, while Spectronaut reports them as 0. The converters in *MSstats* v4.0 automatically interpret the missing values, while taking into account the details of the upstream spectral processing tool, and perform time-consuming (and potentially error-prone) data-preprocessing for the user. Beyond missing values, Data set 2 (DIA) had more peaks than Data set 1 (DDA). The distributions of log2-intensities of the peaks, for example the medians of log2-intensities, were different for both data sets and tools. These differences are not necessarily problematic for peptide and fragment ions that fall in the linear regime of the dynamic range, as similar conclusions regarding changes in protein abundance can be reached from different intensity values. However, they do have important implications for low-abundant analytes.

To maximize the between-tools consistency of the analysis for low-abundant analytes *MSstats* v4.0 learns, separately for each experiment and tool, a threshold for “high-confidence” log_2_-intensities (Step 4 of Figure 2). The threshold is a tuning parameter, defined as the 0.1th percentile of the log_2_-intensities in the linear regime of the dynamic range, and estimated as follows. Define *q*_*p*_ the *p*th percentile of all the log_2_-intensities that exceed 0. In particular, the median is *q*_50_, the 25th percentile is *q*_25_, and the 75th percentile is *q*_75_ (dotted lines in Figure 3). *MSstats* v4.0 estimates *q̂*_0.1_ as
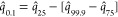
1(dashed lines in Figure 3). The estimation assumes a symmetric distribution of the log_2_-intensities in the linear regime of the dynamic range, and uses *q̂*_99.9_ – *q̂*_75_ to learn the deviation *q̂*_25_ – *q̂*_0.1_.

*MSstats* v4.0 views all “NA” and all the values below *q̂*_0.1_ as censored, i.e., unreliable or missing due to the low abundance of the underlying analyte, with two exceptions. The first exception is Skyline, which reports low-intensity values, and uses “NA” for intensities of truncated or overlapped peaks. For Skyline, *MSstats* v4.0 views “NA” as not associated with low-abundant analytes, and assumes that they are missing at random. The second exception is “NA” reported by any data processing tool for the intensities of reference peptides, e.g., in SRM experiments that use labeled references, or for any other standards. Since reference peptides are not expected to be low abundant, their intensities are also viewed as missing at random, and kept as “NA” by *MSstats* v4.0. This step is optional in the *MSstats* v4.0 workflow.

#### Split-Plot Approach Provides Robust and Accurate Statistical Analysis for Diverse Experimental Designs

In this section, we discuss statistical modeling and analysis in the case of a label-free experiment with group comparison design. A detailed explanation of the overall modeling and analysis workflow, and it extension to experiments with complex designs, can be found in Supporting Information Section 1. This includes extensions to experiments with technical replicates (Supporting Information Section 1.2), time course designs (Supporting Information Section 1.3), paired designs (Supporting Information Section 1.4), and experiments with reference peptide designs (Supporting Information Section 1.5). Finally, a detailed method comparison between *MSstats* v2.0 and v4.0 is available in Supporting Information Section 2.

**Figure 4:**
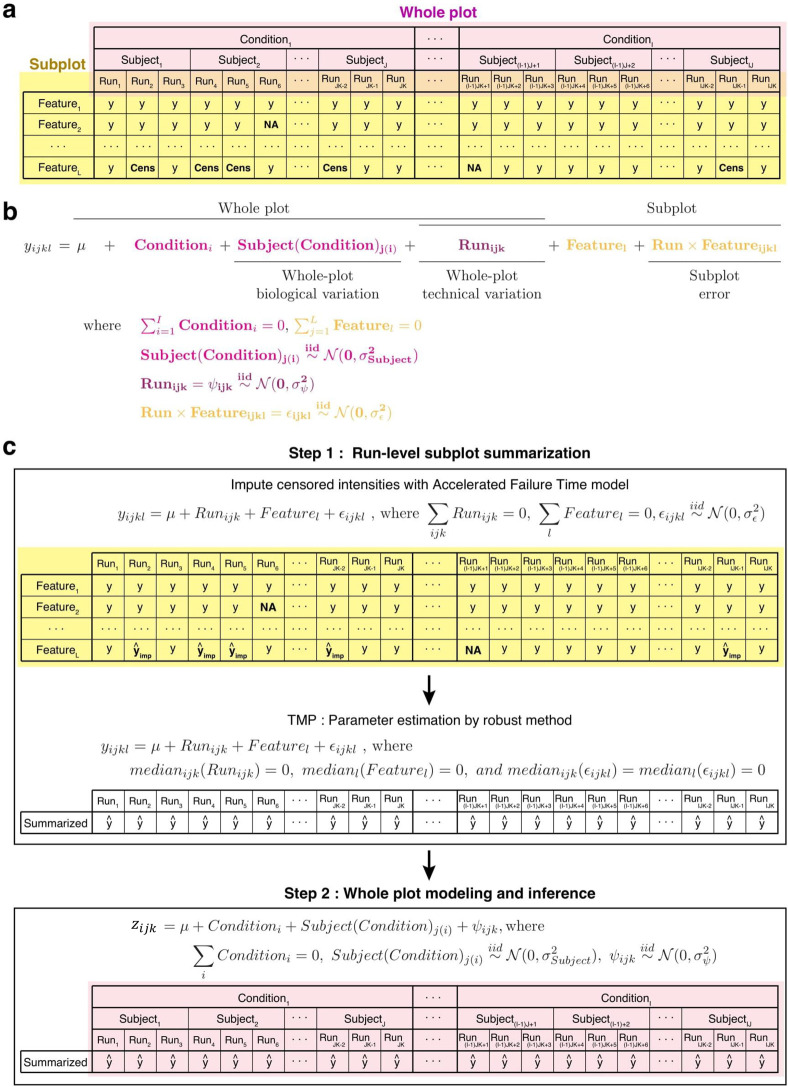
Two-step estimation and inference procedure for the linear mixed-effects model in *MSstats* v4.0, Step 4 of Figure 2. (a) Overview of an example group comparison design. The whole plot and subplot subsections are highlighted. Observed values are indicated as *y*, missing values as NA, and censored values as Cens. (b) The full linear model with the whole plot and subplot sections highlighted. c) The two-step modeling procedure of *MSstats* v4.0. First feature level data are summarized into a single value per run using Tukey’s Median Polish. Next a linear mixed effects model is fit using the summarized values.

Figure 4(a) illustrates the structure of the data for one protein in a label-free experiment with a group comparison design and technical replicates. The experiment has *i* = 1, ..., *I* conditions, e.g., healthy and disease. Each condition is represented by *j* = 1, ..., *J* subjects, i.e., distinct biological replicates (e.g., patients, mice, etc). Subjects are main experimental units, and in this special case of group comparison designs subjects are *nested* within conditions (i.e., each condition is represented by different biological subjects). Furthermore, each subject sample is profiled in *k* = 1, ..., *K* mass spectrometry *Runs*. In practice the number of biological and technical replicates varies across conditions and subjects.

In each run the protein is represented by *l* = 1, ..., *L* spectral features. The features are peptide ions in DDA experiments, combinations of peptide ions and transitions in SRM experiments, and combinations of peptide ions and fragments in DIA experiments. For the purposes of this manuscript we do not distinguish transitions or fragments generated by a same or different peptides. Each feature in each run is quantified by its *Intensity*, *y*_*ijkl*_ (defined as peak area, peak height at apex, or any other measure used by a data processing tool), that are log_2_ transformed and normalized. Such layouts are known in statistical literature as split-plot experimental designs.^41^ More details can be found in Supporting Information Section 1.1.

Figure 4(b) shows a classical split-plot linear mixed effects model of a label-free group comparison experiment with both biological and technical replicates, reflecting the sources of variation in Figure 4(a). In the special case of a balanced experiment with no missing values, these sources of variation are estimated from the analysis of variance (ANOVA) table (Supporting Information Section 1). Unfortunately, the ANOVA decomposition does not hold in experiments with unbalanced designs, censored, and outlying values. An alternative approach to estimating the sources of variation is *restricted maximum likelihood* (*REML*). Unfortunately, in models with many terms such as in Figure 4(b), REML-based estimates can be inaccurate, especially when some sources of variation are close to zero.

As a solution, *MSstats* v4.0 separates the estimation procedure into two simpler steps, namely ANOVA-style summarization in the subplot, and REML estimation in the whole plot as shown in Figure 4(c), as shown in Step 4 of Figure 2. At the whole plot level the summarized data structure has fewer irregularities, and the model has fewer terms. This results in a more stable REML-based estimation. Below we detailed the two-step modeling workflow of the estimation procedure.

#### Modeling Step 1: Subplot Summarization

##### Missing Value Imputation

As an option, *MSstats* v4.0 imputes censored peak intensities (i.e., intensities assumed missing for reasons of low abundance) before subplot summarization. The peak intensities are considered censored if their values are below the cutoff in eq 1 or marked as “NA” (except in the case of Skyline, and in the case of reference peptides and standards). In presence of censored observations, the observed log_2_-intensities *y*_*ijkl*_ are viewed as *y*_*ijkl*_ = *max*(*y*_*ijkl*_, *m*_*ijkl*_), where *m*_*ijkl*_ is the minimum threshold, i.e. the lowest quantifiable log_2_-intensity for that feature. Here we assume that the threshold is feature-specific but constant across the runs, i.e., *m*_*ijkl*_ = *m*_*l*_, and estimate it by the smallest observed log_2_-intensity of the feature. We define an indicator of whether the peak was detected and quantified as
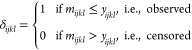
2For the imputation, *MSstats* v4.0 relies on an accelerated failure time (AFT) model^42,43^
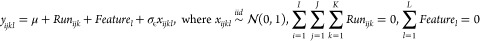
3

The parameters μ, *Run*_*ijk*_, *Feature*_*l*_, and σ in eq 3 are estimated by maximizing the product of the likelihoods of the observed and censored peaks
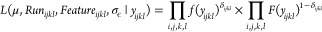
4where *f* is the probability density function and *F* is the cumulative density function of the Normal distribution with expected value μ + *Run*_*ijk*_ + *Feature*_*l*_ and variance σ_ϵ_^2^. The imputed log_2_-intensities are
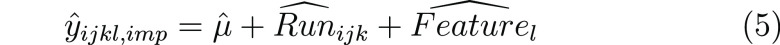
5Therefore, feature imputation is only possible for feature *y*_*ijkl*_ in *Run*_*ijk*_ if there is an observed value for the feature in another run and if there is an observed value from another feature in *Run*_*ijk*_. In particular, features are not imputed if the protein is entirely missing in a run.

**Figure 5:**
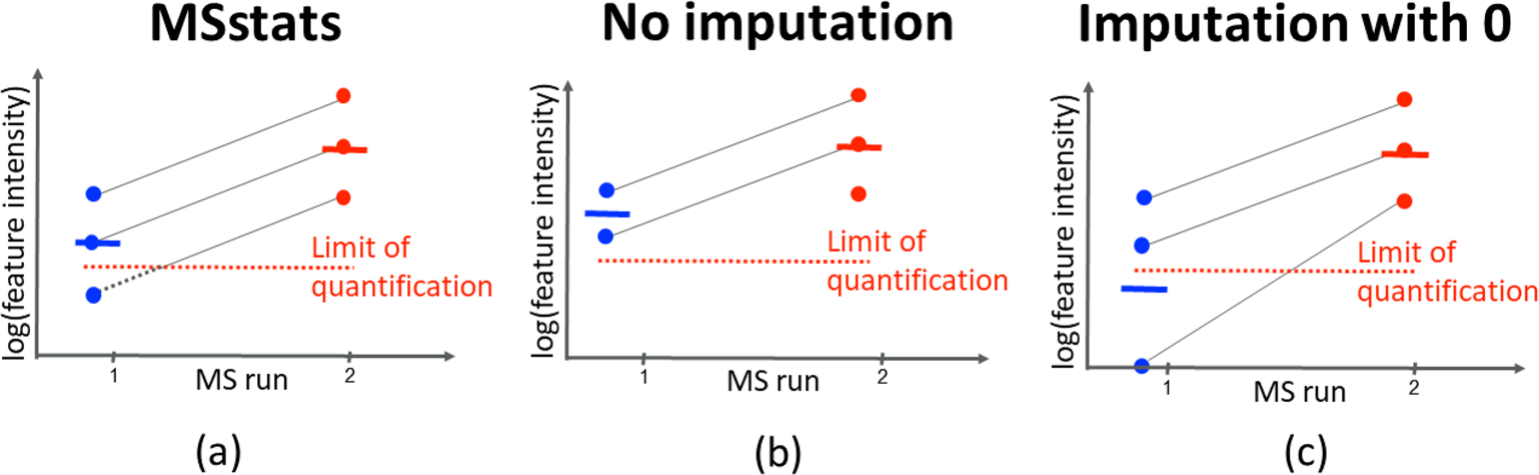
Missing value imputation in *MSstats* v4.0. The plot illustrates a single protein with three features measured over two MS runs. The MS runs are shown on the *x*-axis and the log feature intensity is on the *y*-axis. In the first MS run, colored blue, one of the feature points falls below the limit of quantification and is not measured by the mass spectrometer. (a) *MSstats* imputation. (b) No imputation. (c). Imputation with a small constant.

Figure 5 visualizes the imputation, and contrasts it to other simpler methods. The imputation in eq 4 leverages information from the noncensored values of the feature and the other features in the protein, and relies on the assumption of parallel profiles of features from a same protein between the runs. In contrast, avoiding imputation underestimated the difference between the MS runs, while imputing with a small constant overestimated the difference.

*MSstats* v4.0s imputation of censored values excels for proteins with many features, whose information can be leveraged by the statistical model. In situations with low feature counts, such as when modeling peptides instead of proteins, there may not be enough information to perform imputation and the imputation may not be reliable. As with all imputation methods, *MSstats* imputation relies on the underlying assumption it is making (that values are missing for reasons of low abundance). The performance of *MSstats* missing value imputation can be assessed by looking at the feature-level data after summarization (obtained using the *dataProcess*() function). We recommended that users inspect the modeling assumptions and the resulting imputed values. If the assumptions are violated, for instance if the values are missing at random, the imputation may be biased, and it is best to omit this option.

##### Robust Summarization

We consider an additive model similar to eq 3
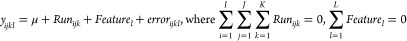
6where μ is the median log_2_-intensity across features and runs, the medians of *Run*_*ijk*_, *Feature*_*l*_, and *error*_*ijkl*_ are centered at 0, and the errors are independent. The difference from eq 3 is that *y*_*ijkl*_ are now both observed and imputed log_2_-intensities, and the parameters of this model are estimated with robust Tukey’s Median Polish (TMP)^38^ that accounts for outlying observations. TMP iteratively subtracts medians of each row (*Feature*) and column (*Run*) from *y*_*ijkl*_ until there is no change. The values remaining in the table after these operations are the residuals of the fit. The run-level summaries  are obtained by subtracting the residuals from *y*_*ijkl*_ and summing the resulting values in the run.

#### Modeling Step 2: Whole Plot Modeling and Inference

At the whole plot level, the linear mixed-effects model is shown in Step 2 of Figure 4(c). In the simple case of a group comparison experiment with biological replicates and no technical replicates, the model is substituted with
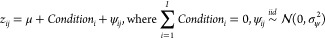
7where σ_ψ_^2^ represents a combination of the biological and the technological variation. The same model is fit for controlled mixtures with technical replicates and no biological replicates, in which case σ_ψ_^2^ is the technological variation. Extensions to complex designs are in (Supporting Information Section 1.

#### *MSstats* v4.0 Provides a Flexible Framework for Comparing Conditions

*MSstats* v4.0 implements a flexible framework for model-based pairwise comparisons between conditions, as well as any linear combinations of conditions such as averaging of multiple groups. To compare the conditions, we first estimate their expected values. The expected value for condition *i* is defined as μ_*i*_ = μ + *Condition*_*i*_, where μ and *Condition*_*i*_ are parameters of the model in Eq. 7. The parameters are estimated from the experimental data using restricted maximum likelihood (REML) (Supplementary Sec. 2.1).

Next we spell out the comparison of interest, which involves two or more expected values.^44,45^Eq. 8 defines a linear combination *l* of the expected values μ_*i*_.
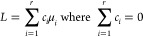
8For example, a pairwise comparison between the expected values of Conditions 1 and 2 is expressed with coefficients *C* = (1, −1, 0, 0). As another example, a comparison between the average of the expected values of Conditions 1 and 2 versus the average of the expected values of Conditions 3 and 4 is expressed with coefficients *C* = (1/2, 1/2, −1/2, −1/2). The special cases of linear combinations, where the coefficients *c*_*i*_ sum to zero, are called a contrasts. *MSstats* v4.0 can estimate any linear combination of the expected values, not just a contrast. To test the null hypothesis *H*_0_: *L* = 0 against the alternative *H*_*a*_: *L* ≠ 0, the estimate of the linear combination *L̂* and its standard error are combined into a *t*-statistic
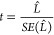
9which is then compared against the student distribution with appropriate degrees of freedom to determine the *p*-value.^44^

We now illustrate this model-based inference in the special case of an experiment with *I* conditions and *J* biological replicates per condition in a balanced design, the model Figure 4(c), and a pairwise comparison between Conditions 1 and 2. The model expresses the expected values μ_1_, μ_2_, μ_3_, μ_4_ of the four conditions. In the special case of balanced designs and simpler ANOVA-based estimation, the model-based estimate of an expected values matches its sample averages, i.e., μ̂_*i*_ = *z̅*_*i*_.^44^

Since the contrast coefficients of interest are *C* = (1, −1, 0, 0), *L̂* = *z̅*_1_ – *z̅*_2_ is the model-based estimate of the log_2_-fold change between the conditions. In this special case, the standard error of *L̂* is estimated as
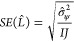
10where σ_ψ_^2^ is the ANOVA-based estimate of the error variance in Figure 4(c). Finally, in this special case, the *t*-statistic in eq 9 is compared against the Student distribution with *I*(*J* – 1) degrees of freedom to determine the *p*-value.

### Implementation

#### New Coding Strategies Improve Memory and Time Computational Complexity

*MSstats* v2.0 and v3.0 exclusively used base R^46^ verbs with parts of code written with tidyverse.^47^*MSstats* v4.0 implements all operations on tabular data with data.table^48^ verbs, which ensures strong performance for large data sets that fit in memory.^49^ In particular, whenever possible, *MSstats* v4.0 relies on grouped tabular operations rather than iterative procedures. Moreover, parts of the code, including summarization and postprocessing of fitted statistical models, were rewritten with C++ using the *Rcpp* interface^50^ to improve execution time. Performance of logging was improved by the addition of log4r backend,^51^ while performance of parameter validity checks relies on the checkmate package.^52^

*MSstats* v4.0 is highly modularized. Code related to preprocessing raw outputs of signal processing tools was moved to the *MSstatsConvert* package, and converters in *MSstats* rely on functions from that package. This backend can be reused to ensure high performance of new and custom converters. Moreover, updated *MSstats* provides a set of alternative functions to the existing *dataProcess*() and *groupComparison*(). With these functions, each step of the *MSstats* workflow is implemented in a separate function (such as *MSstatsNormalize*() for normalization, *MSstatsSummarize*() for summarization) which serve as backend for established functions, but can be used independently. This alternative workflow improves both time and memory management, as follows. First, it is possible to reduce the amount of repeated operations when replicating the analysis. For example, new analysis may start with normalized data without the need to rerun operations done by the *dataProcess*() function before normalization. Second, this workflow was designed with parallel execution in mind. Summarization (particularly with imputation) and model fitting are the most time- and memory-consuming parts of *MSstats* workflow. Both operations are done separately for each protein in a data set. Thus, operations that use all data are implemented with *data.table* verbs for maximum performance, while per-protein operations are provided in self-contained functions (such as *MSstatsSummarizeSingleTMP*()) that can be run in parallel. Due to multiplicity of architectures for parallel computations and differences in parallelization options across operating systems, we do not provide parallel versions of *MSstats* function. Instead, the current version of the package provides tools which allow users to take advantage of their own parallel infrastructure. Moreover, these building blocks of *MSstats* workflow are easily reusable across packages that implement data analysis methods for different experimental workflows.

#### MSstatsBig Enables the Analysis of out of Memory Data Sets

While the *data.table* backend and other updates in *MSstats* v4.0 ensure strong performance for in-memory data sets, handling data larger than memory is a challenge for the *MSstats* workflow. Different steps of the workflow require aggregation across different variables (such as Run and Protein), which makes simple batch-processing and complete reuse of existing code infeasible. Thus, motivated by large data sets, such as the ones generated with Spectronaut, we created *MSstatsBig* currently available on the *MSstats* GitHub page https://github.com/Vitek-Lab/MSstatsBig. The package implements a restricted (in terms of freedom of parameter choice) version of *MSstats* workflow, assuming that feature-level input data do not fit in the memory but preprocessed data in *MSstats* format do. Since the *MSstats* preprocessing removes low-quality and redundant PSMs, and aggregates measurements repeated for a given Run and Feature, there are many opportunities for reducing data set. Further reduction in data size can be achieved by reducing redundancy in Run annotation or changing Protein/Feature labels.

The biggest challenge in processing out-of-memory data sets are grouped operations, as grouping does not necessarily correspond to physical partitioning of the data. Thus, we use SparklyR package to connect to Apache Spark database to process data. This allowed us to process data set as big as 200 GB.

## Evaluation

### Evaluation Criteria

We compared the performance of *MSstats* v4.0, v3.0, and v2.0 in terms of computational time. To test computation speed, we first converted the data into the required format, using the corresponding converter for v4.0 and v3.0, and manually for v2.0, and ran the full statistical workflow for each version, using the *dataProcess*() and *groupComparison*() functions. The functions were each run 5 times for each version and each data set and the mean run time for the workflow was recorded.

We compared the statistical results of *MSstats* v2.0, *MSstats* v4.0, *MSqRob*, and *DEqMS* on the controlled mixtures and biological experiments. For *MSstats* v4.0, the raw files were input into the corresponding converters, and the default parameters were used for the *dataProcess*() and *groupComparison*() functions. For *MSqRob* the data were first manually converted into the required format, and then the workflow noted in the *MSqRob*vignette was followed with default parameters selected. For *DEqMS* the data were manually converted and the vignette workflow was generally followed. However, because the summarization function requires TMT labeled data, we manually summarized the feature level data using the log sum of features.

For statistical results, the versions were evaluated on the controlled mixtures (data sets 1, 2, and 3) in terms of true positive (TP), true negative (TN), false positive (FP), and false negative (FN) differentially abundant proteins while controlling the FDR at 5%. The true positives were defined as spike-in proteins with known changes in abundance. The true negatives were defined as spike-in proteins with no changes in abundance. Additionally, the positive predictive value (PPV)/empirical False Discovery Rate (eFDR) was calculated as described in eq 11.
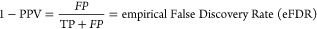
11For the biological experiments (data sets 4 and 5) without known ground truth, we compared the lists of differentially abundant proteins produced by each version.

### Evaluation

#### *MSstats* v4.0 Reduced Processing Time and Memory Consumption Across All Data Sets

Table 5 reports the computational resources used for *MSstats* versions 2.0, 3.0, and 4.0. *MSstats* v4.0 drastically reduced the mean processing time across all data sets (an average decrease of 74.66%). The processing time was dramatically reduced due to both changes in modeling strategy and refactoring of the code base. Memory allocation reports the line profiling for the complete statistical workflow (summarization and group comparison). Across all data sets both the total and maximum allocated memory decreased with version 4.0. The maximum allocation was stable despite different characteristic of the data (including data acquisition mode). The decrease in allocation ranged from about 10% to 65%, depending on a data set. This change was mainly due to technological and code improvements from v3 to v4. Table 5 shows the reduction in memory usage was inconsistent from v2.0 to v3.0, however there was a drastic reduction from v3.0 to v4.0.

**Table 5:** Use of Computational Resources by *MSstats* v2.0, v3.0, and v4.0 on Each Data Set^a^

		Processing Time [s]			Mem. Total [MB]			Mem. Sum Max [MB]		
Data Set	File Size	v2	v3	v4	v2	v3	v4	v2	v3	v4
1: Controlled Mixture–DDA–MaxQuant	200 MB	831.56s	231.77s	164.38s	16,583.60	40,315.09	13,542.00	109.26	147.24	20.21
2: Controlled Mixture–DIA–Spectronaut	1.04 GB	3,895.67s	1,428.82s	963.18s	20,726.46	17,920.83	7,090.18	291.09	243.60	13.76
3: Controlled Mixture–DDA–Skyline	63 MB	243.52s	97.10s	42.35s	20,341.49	15,176.89	6,042.41	100.64	111.61	20.54
4: Mouse–DDA–MaxQuant	257 MB	1,076.97s	636.60s	338.98s	16,948.49	24,507.43	10,665.79	297.54	335.40	16.17
5: *S. cerevisiae*–DIA-Skyline	315 MB	4,411.80s	2,258.34s	1,897.62s	85,845.08	26,531.06	18,125.68	412.43	234.18	29.83

a “Processing time” is the time in seconds the data sets took to run. *MSstats* versions 2.0, 3.0, and 4.0 were each measured 5 times per data set, and the mean processing time was reported. Because there was no converter in version 2.0, we applied data conversion to the experiments before measuring processing time. Measurement was done using the microbenchmark package.^53^ “Mem. total [MB]” is the total memory allocation for summarization and group comparison across controlled mixtures and biological experiments. “sum of maximum allocation” is the sum of maximum allocation for summarization and group comparison across all data sets. *MSstats* v2.0, v3.0, and v4.0 were run once for each data set and memory allocation was reported for *dataProcess* and *groupComparison* step. Here, we report total allocation for both steps of the workflow and sum of maximum allocation in both steps. Measurement was done using the lineprof package.^54^

#### Statistical Methods in *MSstats* v4.0 Improved eFDR in Three Controlled Mixtures

**Figure 6:**
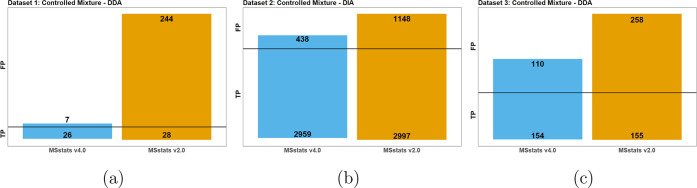
Controlled data sets 1, 2, and 3: Statistical analyses by *MSstats* v2.0 and v4.0. The true positives (TP) and false positives (FP) reported by each version. The FP are shown on the top, and the TP are shown at the bottom of each bar, with the black line showing 0. In all data sets, the TP numbers were similar between versions, while the FP were much lower when using v4.0. (a) Data set 1: Controlled Mixture–DDA–MaxQuant. (b) Data set 2: Controlled Mixture–DIA–Spectronaut. (c) Data set 3: Controlled Mixture–DDA–Skyline.

Figure 6 reports the results of applying *MSstats* v2.0 and v4.0 to the three controlled mixtures. In all the data sets the number of TP were nearly equal, however the number of FP markedly decreased. Subsequently the eFDR in Data set 1 decreased from 89.7% in v2.0 to 20.6% in v4.0, the eFDR in Data set 2 decreased from 27.7% to 12.9%, and the eFDR in Data set 3 decreased from 62.45% to 41.67%. *MSstats* v2.0 overestimated the number of positives in all cases, resulting in a much higher eFDR.

#### *MSstats* v4.0 Improved Standard Error and Degrees of Freedom Calculation in Biological Experiment

The difference in performance of the versions of *MSstats* was mainly caused by differences in statistical modeling and model fitting. *MSstats* v2.0 reported overly high degrees of freedom at the pairwise comparison stage, and as the result was overly sensitive, and detected differentially abundant proteins even when there was no true change between conditions. The whole plot model used by *MSstats* v4.0 summarized all features into a single value per MS run prior to fitting the final model. This facilitated the estimation, and resulted in a more appropriate estimation of variability and degrees of freedom.

**Figure 7:**
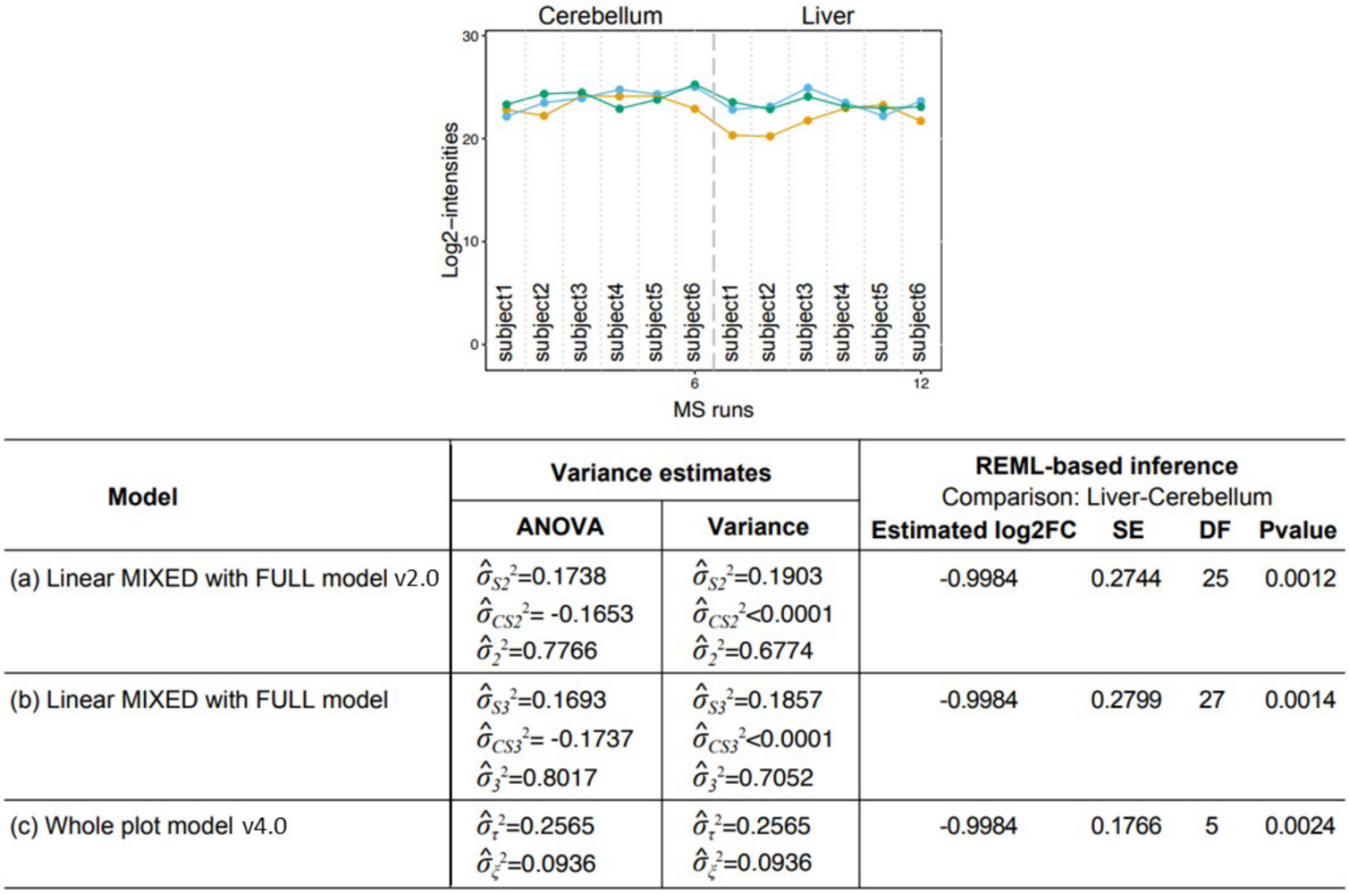
Data set 4: Mouse–DDA - MaxQuant, Protein O08547 In this example, the estimate σ̂_*CS*_^2^ of the Condition × Subject was zero, and this severely undermined the estimation of degrees of freedom in *MSstats* v2.0 and in the full model v4.0. In contrast, the whole plot model in *MSstats* v4.0 was simpler, had fewer variance components, and the variance components were away from zero. This produced a smaller estimate of standard error(SE) of the pairwise comparison and the on-target degrees of freedom. After adjusting for multiple comparisons, such difference can affect the decision of differential abundance.

Figure 7 illustrates a protein in Data set 4: Mouse - DDA - MaxQuant where REML estimation had a small impact on pairwise comparisons between conditions. The protein had a balanced design, and the models in *MSstats* v2.0 and v4.0 had identical theoretical ANOVA-based inference. In this example, the estimate σ̂_*CS*_^2^ of the Condition × Subject was zero, and this severely undermined the estimation of degrees of freedom in *MSstats* v2.0 and in the full model v4.0. In contrast, the whole plot model in *MSstats* v4.0 was simpler, had fewer variance components, and the variance components were away from zero. This produced a smaller estimate of standard error of the pairwise comparison, and the on-target degrees of freedom. After adjusting for multiple comparisons, such difference can affect the decision of differential abundance. x

#### *MSstats* v4.0 Better Traded off False Positives and False Negatives and Had an Easier Use than *MSqRob* and *DEqMS* in Controlled Mixtures

**Figure 8:**
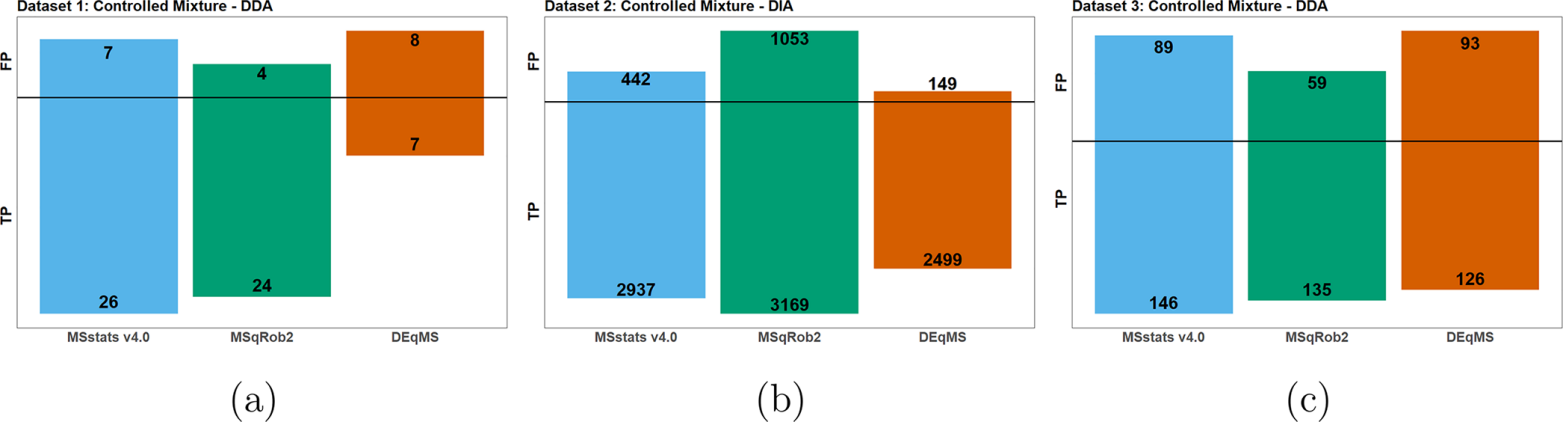
Controlled data sets 1, 2, and 3: Statistical analyses by *MSstats* v4.0, *MSqRob*, and *DEqMS*. The true positives (TP, below the horizontal line) and false positives (FP, above the horizontal line) reported by each tool. (a) Data set 1: Controlled Mixture–DDA–MaxQuant. The results between *MSstats* and *MSqRob* were similar, with *MSstats* being a more sensitive, detecting more true positives and false positives, while *DEqMS* lagging behind. (b) Data set 2: Controlled Mixture–DIA–Spectronaut. *DEqMS* was less sensitive than the other methods, missing many true positives but reporting few false positive. *MSstats* reported fewer false positives than *MSqRob* but also did not identify as many true positives. (c) Data set 3: Controlled Mixture–DDA–Skyline. All three methods were comparable. *MSstats* reported the most TP but also reported more FP than *MSqRob*.

Figure 8 reports the results of the controlled mixtures using *MSstats* v4.0, *MSqRob*, and *DEqMS* in terms of TP and FP. In all data sets, *DEqMS* exhibited the poorest performance, mostly due to lack of dedicated data preprocessing functionality. Both *MSstats* v4.0 and *MSqRob* implement multiple data preprocessing steps directly into their workflows, and have dedicated protein summarization functions. In contrast, *DEqMS* required manual implementation of the majority of these steps. As the result, the feature-level measurements included low quality features, and the summarization was not as robust as *MSstats* and *MSqRob*’s summarization. This in turn produced worse statistical results.

*MSstats* v4.0 and *MSqRob* performed similarly on all controlled mixtures. In data sets 1 and 3 *MSstats* reported a higher number of TP and FP than *MSqRob*, whereas in data set 2 *MSqRob* reported more TP and FP. While the statistical results were similar, the application of *MSstats* to the data sets was much more straightforward. The output of MaxQuant and Spectronaut could be directly converted into *MSstats* format using the dedicated converters, whereas the data for *MSqRob* had to be manually converted. The data preprocessing and summarization for *MSstats* was applied using one function (*dataProcess*()) with all options laid out as function parameters. In comparison, *MSqRob* required each preprocessing step to performed separately, with some processing steps, such as filtering nonzero intensities, requiring manual implementation. Additionally, in data set 1 *MSqRob* reported uninformative errors due to features entirely missing in some conditions. These features had to be manually filtered out in order for the summarization function to complete. In contrast, *MSstats* automatically took care of these features and required no extra work or debugging by the user.

#### *MSstats* v4.0 Identified New Differentially Abundant Proteins As Compared to *MSqRob* and *DEqMS* in Biological Experiments

**Figure 9:**
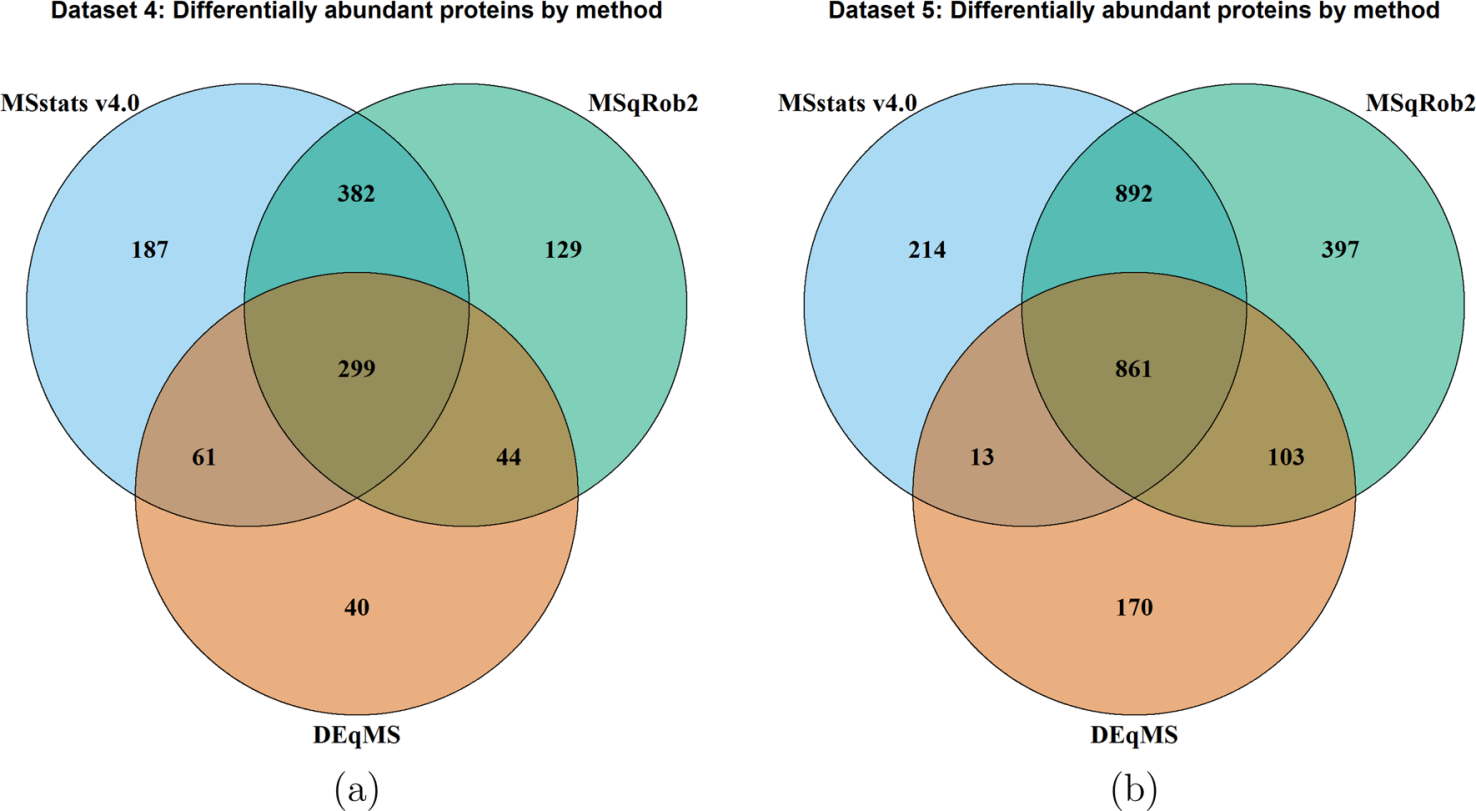
Data sets 4 and 5: Statistical analyses by *MSstats* v4.0, *MSqRob*, and *DEqMS*. (a) Data set 4: Mouse–DDA–MaxQuant. *MSstats* and *MSqRob* show a large overlap of differentially abundant proteins, with *MSstats* reporting more differentially abundant proteins in total. *DEqMS* produced the least differentially abundant proteins, with most of those reported also reported by the other methods. (b) Data set 5: *S. cerevisiae*–DIA–Skyline. *MSstats* and *MSqRob* show a large overlap of differentially abundant proteins, however *MSqRob* reported more differentially abundant proteins in total than *MSstats*.

Figure 9 shows the number of differentially abundant proteins reported by each method and their overlap for biological experiments in this manuscript. Unlike the controlled mixtures, the experiments contain biological variation, and represent repeated measures design. Consistently with the previous section, *MSstats* v4.0 and *MSqRob* reported the highest number of the differentially abundant proteins. In Data set 4, *MSstats* reported 929 differentially abundant proteins, while *MSqRob* reported 854. In Data set 5 *MSstats* reported 1980 differentially abundant proteins and *MSqRob* reported 2253. These two methods had a large overlap in both data sets. In Data set 4 there was an overlap of 681 differentially abundant proteins, 51% of all differential proteins being reported by any method. In Data set 5, there was an overlap of 1753, 66% of all differential proteins. While the results between these methods were similar, *MSstats* was much easier to apply. It required only 8 lines of code, calling data converter and wrapping all the data preprocessing functionality. *MSqRob* on the other hand was much more complicated to apply, taking 60 lines of code to complete the analysis. Additionally, since the experiments had repeated measures design the models in *MSqRob* had to be entered manually, which can be challenging for users with limited statistical expertise.

*DEqMS* reported the least number of differential proteins, only 444 in Data set 4 and 1147 in Data set 5 (less than half of those reported by *MSstats* and *MSqRob*). The majority of proteins reported by *DEqMS* were also reported by the other methods. As described previously, this is most likely due to the lack of data preprocessing by the *DEqMS* workflow.

#### MSstatsBig Enabled Analysis of out-of-Memory Data

We preprocessed both out of memory simulated data sets using *MSstatsBig* functionalities: *cleanBigSpectronaut*() function that performs initial data reduction and saves intermediate result which can be processed with standard *MSstatsConvert* tools or using the *BigSpectronauttoMSstatsFormat*() function from *MSstatsBig*. Execution time was measured using *system.time* function from base R. Table 6 summarizes the results. *Processing* step refers to the *cleanBigSpectronaut*() function while *Cleaning* step is implemented by *BigSpectronauttoMSstatsFormat*(). We used the Arrow^55^ package backend for this benchmark. *MSstatsBig* package also supports cleaning data with dplyr and sparklyr backends.

**Table 6:** Use of Computational Resources in out-of-Memory Analyses with *MSstats* v4.0^a^

		Elapsed Time [s]		
Number of Copies	File Size	Processing	Cleaning	Total
15	17.3 GB	210.75	439.75	650.50
30	34.6 GB	380.54	1351.24	1731.78

a Processing time for two data sets created by merging copies of the Controlled Mixture–DIA. Computations were done a laptop with 16 GB RAM and 2.3 GHz CPU with 4 cores.

## Discussion

The manuscript describes a substantial update to the core package of the *MSstats* Bioconductor family of packages. The updates improved the usability of the implementation, the statistical methodology, and the computational resource requirements. The package now includes converters which directly integrate it with the output of multiple spectral processing tools used for analyte identification and quantification. The converters greatly increase the usability of the package, making it much easier for users to begin their analysis, without having to go through a lengthy manual conversion. The new statistical methods are applicable to a broader range of experimental designs, and improved the accuracy of the statistical inference as compared to the previous version. The new implementation drastically improved both the computational speed and memory usage of the package. The users can now process experiments of size that was out of reach for older versions of *MSstats*.

In comparisons with *MSqRob* and *DEqMS*, *MSstats* v4.0 performed favorably, while being easier to use and implement. The existing methods relied on manual implementation by the user for upstream data processing, and focused mainly on the final statistical model. In contrast, *MSstats* v4.0 encompassed the entire data analysis pipeline, from the output of spectral processing tools, to upstream data processing, and statistical analysis. It included a wide range of additional analysis options, such as plotting functionality to visualize the results of data analysis, without depending on other tools. The broader scope of *MSstats* v4.0 eased the analysis of all data sets in the manuscript, requiring only a few lines of code to go from raw data to group comparison and high quality statistical analysis.

Overall, we believe that *MSstats* v4.0 is a strong contribution to reproducible mass spectrometry-based proteomic research, taking a user-first approach to MS experimental analysis and producing accurate statistical results in a straightforward, easy to apply, implementation.

## Supporting Information Available

Data structure in a quantitative proteomic experiment; example group comparison design; full linear mixed-effects model for one protein; analysis of variance for the model; ANOVA-based pairwise comparison of conditions; example time course design; full linear mixed-effects model for one protein; analysis of variance for the model; ANOVA-based pairwise comparison of conditions; example paired experimental design; data structure for one protein, from an experiment with labeled reference peptides; full linear mixed-effects model for one protein; ANOVA-based inference for pairwise comparisons in balanced label-free designs; ANOVA-based inference for pairwise comparisons in time course or paired label-free designs; ANOVA-based inference for pairwise comparisons in balanced label-free designs with technical replicates only; example annotation file for the group comparison design; example annotation file for the time course design; and example annotation file for the paired design (PDF)
