# Supplementary material for: MSstats Version 4.0: Statistical Analyses of Quantitative Mass Spectrometry-Based Proteomic Experiments with Chromatography-Based Quantification at Scale
Source: J Proteome Res. 2023 Apr 5;22(5):1466–82. doi: 10.1021/acs.jproteome.2c00834 (PMC10629259; doi:10.1021/acs.jproteome.2c00834)
Supplement: Supplementary file 1 — pr2c00834_si_001.pdf [file pr2c00834_si_001.pdf]

MSstats 4.0: statistical analyses of quantitative mass spectrometry-based proteomic experiments with chromatography-based quantification at scale

Supplementary Information

Devon Kohler[1], Mateusz Staniak[2], Tsung-Heng Tsai[1] Ting Huang[1], Nicholas Shulman[3], Oliver M. Bernhardt[5], Brendan X. MacLean[3], Alexey I. Nesvizhskii[6], Lukas Reiter[5], Eduard Sabidó[7,8], Meena Choi[1$*], Olga Vitek[1*]

[1] Khoury College of Computer Sciences, Northeastern University, Boston, MA, USA
[$] Current address: Microchemistry, Proteomics and Lipidomics, Genentech, South San Francisco, CA, USA
[2] University of Wrocław, Wrocław, Poland
[3] Department of Genome Sciences, University of Washington, Seattle, WA, USA
[4] OMNI Biomarker Development, Genentech, South San Francisco, CA, USA
[5] Biognosys, Zürich, Switzerland
[6] Department of Pathology and Computational Medicine & Bioinformatics, University of Michigan, Ann Arbor, MI, USA
[7] Proteomics Unit, Center for Genomics Regulation, Barcelona Institute of Science and Technology(BIST), Barcelona, Spain
[8] Proteomics Unit, Universitat Pompeu Fabra, Barcelona, Spain

∗ Corresponding authors: Meena Choi : choi.meena@gene.com and Olga Vitek : o.vitek@northeastern.edu

Contents

1 Supplementary Note 1 : Detailed description of experimental designs compatible with *MSstats* v4.0 **3**

1.1 Design with intentional restriction on randomization: a split-plot 3

1.2 Extension to label-free group comparison experiment with technical replicates 5

1.2.1 Experimental design 5

1.2.2 Annotation file 5

1.2.3 Statistical details 5

1.3 Extension to label-free time course designs 8

1.3.1 Experimental design 8

1.3.2 Annotation file 8

1.3.3 Statistical details 8

1.4 Extension to label-free paired designs 11

1.4.1 Experimental design 11

1.4.2 Annotation file 11

1.4.3 Statistical details 11

1.5 Extension to group comparison experiment with labeled reference peptides 13

1.5.1 Experimental design 13

1.5.2 Annotation file 13

1.5.3 Statistical details 13

2 Supplementary Note 2 : MSstats v2.0 versus MSstats v4.0 **16**

2.1 Differences in theoretical ANOVA-based inference in balanced designs 16

Supplementary Note 1 : Detailed description of experimental designs compatible with *MSstats* v4.0

1.1 Design with intentional restriction on randomization: a split-plot

An important concept in statistical design and analysis of experiments is *experimental unit*, i.e. a unit to which we apply the randomization of sample selection and data acquisition [1]. A data structure such as in **Figure 1**(a) could in principle be generated by a completely randomized factorial design [2]. The design assumes that the experimental units are cells (i.e., intensities of individual features), and the randomization is applied to these units as described in **Supplementary Fig. 1**(a). According to the example in the figure, we would first acquire the intensity of Feature 1 in Run 5, then Feature 2 in Run JK, then again Feature 1 in Run 3, and so on. This strategy is, of course, both impractical and ineffective.

Instead, proteomic experiments intentionally impose a restriction on randomization, where the randomization is performed at the level of biological samples and runs. The experiment then intentionally restricts the randomization to acquire all the intensities of all the features in a run at once, as described in **Supplementary Fig. 1**(b). The resulting data are affected by unequal variation. The variation between runs represents both biological variation, and technological between-run variation from sources such as sample storage and processing, and changes in instrument performance. On the other hand, all the feature intensities acquired within a same run share common sources of variation that pertain to that sample and that run. They only reflect the between-feature and within-run nuisance variation, which is typically smaller.

In statistical literature the layout in **Supplementary Fig. 1**(b) and in **Figure 1**(a) is called *split-plot design*. The source of biological and of between-run technological variation is called *whole plot*. The source of between-feature, within-run nuisance variation is called *subplot*. The design, and approaches to the analysis of data with this design, are well documented [2]. MSstats leverages the properties of this design to improve detection of differentially abundant proteins in proteomic experiments with diverse input data and experimental designs.

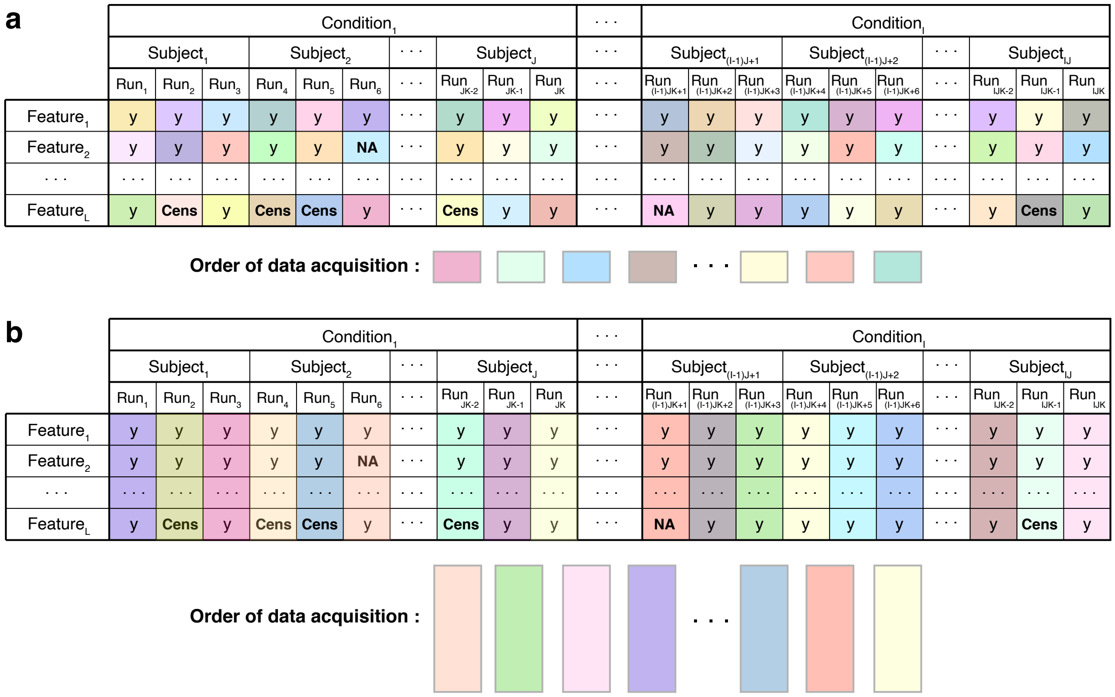

Supplementary Fig. 1: **Data structure in a quantitative proteomic experiment.** Colors indicate experimental units to which randomization is applied. (a) Completely randomized factorial design. It is impractical and ineffective in proteomics. (b) Split-plot design. It reflects the actual process of data collection.

1.2 Extension to label-free group comparison experiment with technical replicates

1.2.1 Experimental design

In **Statistical Methods and Implementations in MSstats v4.0** we reviewed *MSstats* v4.0 as applied to a group comparison design without technical replicates. Here we extend this model to an experiment with multiple technical replicates per biological replicate. A technical replicate is a separate run using the same biological material. **Supplementary Fig. 2** details a group comparison design with 2 conditions, 3 biological replicates per condition, and 2 technical replicates per biological replicate, resulting in 12 total MS runs. **Figure 4** shows this design in tabular format, and expands it to include any number of replicates and conditions.

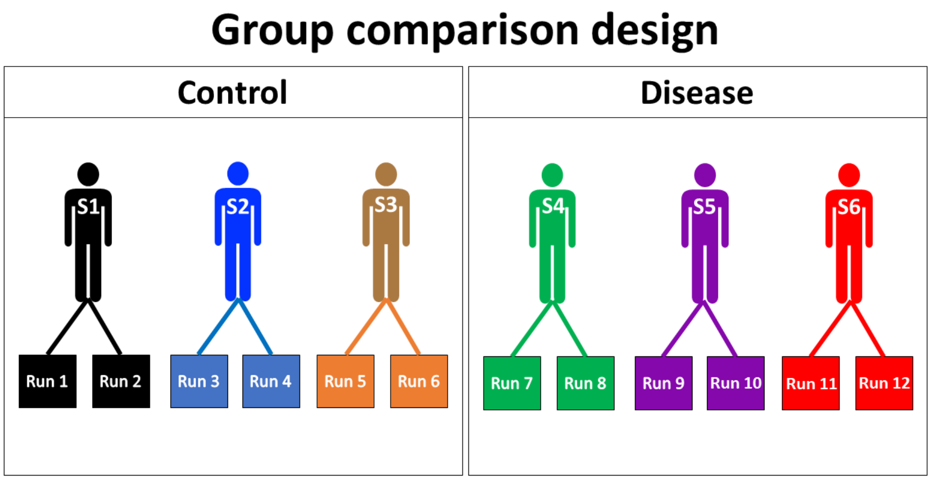

Supplementary Fig. 2: **Example group comparison design.** 12 total MS runs spread across 2 conditions, 3 biological replicates per condition, and 2 technical replicates per biological replicate. Each biological replicate only belongs to one condition; either the control or disease.

1.2.2 Annotation file

MSstats v4.0 requires the user encode their experimental design in an "annotation" file which details each condition, biological replicate, and MS run in the experiment. *MSstats* v4.0 will use this file to learn the experimental design and automatically adjusts the underlying models accordingly. **Supplementary Table 1** details an example annotation file for the experiment in **Supplementary Fig. 2**. The order of biological replicates and conditions in the experiment should be randomized, and the run id does not need to represent the order of spectral acquisition. Additionally, the values of the columns are repeated for every quantified transition. Note that technical replicates will automatically be inferred by *MSstats* and are not required in the annotation file.

1.2.3 Statistical details

Supplementary Fig. 3 shows a classical full linear mixed effects model for a split-plot representation of a label-free group comparison experiment, with both biological and technical replicates. The model reflects the sources of variation in **Figure 4**. In the special case of a balanced experiment with no missing values, these

Condition	BioReplicate	Run
Control	S1	Run 1
Control	S1	Run 2
Control	S2	Run 3
Control	S2	Run 4
Control	S3	Run 5
Control	S3	Run 6
Disease	S4	Run 7
Disease	S4	Run 8
Disease	S5	Run 9
Disease	S5	Run 10
Disease	S6	Run 11
Disease	S6	Run 12

Supplementary Table 1: **Example annotation file for the group comparison design in Supplementary Fig. 2**. The run names must match exactly with the run names used in the data generated by the upstream data processing tool. Note technical replicates are not required in the annotation file. *MSstats* will automatically detect that the biological replicates are repeated twice per condition and will adjust the model appropriately.

sources of variation are estimated from the analysis of variance (ANOVA) table in **Supplementary Fig. 4**. The table shows that different error terms should be used when comparing conditions, subjects and features. Since *MSstats* v4.0 focuses on comparing conditions, **Supplementary Fig. 5** shows that estimated fold changes and their variances do not rely on the individual $\log_2$-intensities, but on a summary (i.e., average) of $\log_2$-intensities in a run. Unfortunately, the ANOVA decomposition does not hold in experiments with unbalanced designs, censored, and outlying values. *MSstats* v4.0 extends the summary-based inference procedure to these more general situations.

$$y_{ijkl} = \mu + \overbrace{\mathbf{Condition}_i + \underbrace{\mathbf{Subject(Condition)_{j(i)}}}_{\text{Whole-plot biological variation}} + \underbrace{\mathbf{Run_{ijk}}}_{\text{Whole-plot technical variation}}}^{\text{Whole plot}} + \overbrace{\mathbf{Feature}_l + \underbrace{\mathbf{Run \times Feature_{ijkl}}}_{\text{Subplot error}}}^{\text{Subplot}}$$

where

$$\sum_{i=1}^{I} \mathbf{Condition}_i = 0,\ \mathbf{Subject(Condition)_{j(i)}} \overset{\text{iid}}{\sim} \mathcal{N}(\mathbf{0}, \sigma^{\mathbf{2}}_{\mathbf{Subject}}),\ \mathbf{Run_{ijk}} \overset{\text{iid}}{\sim} \mathcal{N}(\mathbf{0}, \sigma^{\mathbf{2}}_{\mathbf{Run}}),$$

$$\sum_{j=1}^{L} \mathbf{Feature}_l = 0,\ \mathbf{Run \times Feature_{ijkl}} = \epsilon_{\mathbf{ijkl}} \overset{\text{iid}}{\sim} \mathcal{N}(\mathbf{0}, \sigma^{\mathbf{2}}_{\epsilon})$$

Supplementary Fig. 3: **Full linear mixed-effects model for one protein in Supplementary Fig. 2** y_{ijkl} is the $\log_2$-intensity of the ith *Condition*, jth *Subject*, kth *Run*, and lth *Feature*. μ is the mean $\log_2$-intensity of the protein. The model decomposes the variation in the $\log_2$-intensities into contributions from each source. Pink: sources of variation in the whole plot. Yellow: sources of variation in the subplot.

Model term	Sum of squares (SS)	Degrees of freedom	Expected mean squares
Condition	$JKL\sum_i(\bar{y}_{i...} - \bar{y}_{....})^2$	$I-1$	σ^2_ϵ + Lσ^2_{Run} + KL$\sigma^2_{Subject}$ + $\frac{JKL\sum Condition_i^2}{I-1}$
Subject(Condition)	$KL\sum_{ij}\bar{y}_{ij..} - \bar{y}_{i...})^2$	$I(J-1)$	σ^2_ϵ + Lσ^2_{Run} + KL$\sigma^2_{Subject}$
Wholeplot error (Run)	$L\sum_{ijk}(\bar{y}_{ijk.} - \bar{y}_{ij..})^2$	$IJ(K-1)$	σ^2_ϵ + Lσ^2_{Run}
Feature	$IJK\sum_l(\bar{y}_{...l} - \bar{y}_{....})^2$	$L-1$	σ^2_ϵ + $\frac{IJK\sum Feature_l{}^2}{L-1}$
Subplot error (R×F)	$\sum_{ijkl}(y_{ijkl} - \bar{y}_{ijk.} - \bar{y}_{...l} + \bar{y}_{....})^2$	$(IJK-1)(L-1)$	σ^2_ϵ
Total	SS_{Total}	$IJKL-1$	

Supplementary Fig. 4: **Analysis of variance for the the model in Supplementary Fig. 3, in the special case of balanced designs.** Horizontal line separates whole plot and subplot, which have a different structure of random error. Gray box indicates the error term relevant when comparing conditions.

Pairwise comparison	Estimated log-fold change	Theoretical variance	Estimated variance	Degrees of freedom
Condition$_i$ − Condition$_{i'}$	$\bar{y}_{i...} - \bar{y}_{i'...}$	$\frac{2\sigma^2_\epsilon}{JKL} + \frac{2\sigma^2_{Run}}{JK} + \frac{2\sigma^2_{subject}}{J}$	$\frac{2}{JKL} \times \frac{SS_{Subject(Condition)}}{I(J-1)}$	$I(J-1)$

Supplementary Fig. 5: **ANOVA-based pairwise comparison of conditions, based on Supplementary Fig. 4.** *SS* indicates sum of squares.

1.3 Extension to label-free time course designs

1.3.1 Experimental design

In label-free experiments with time course designs, the subplot structure is the same as in a group comparison design. The whole plot differs from a group comparison design, as it contains repeated measurements on a same biological replicate, across *Time* (*Condition* represents *Time* in time course experiments). **Supplementary Fig. 6** details an example time course experiment with 2 biological replicates spread across 3 different time points. Each biological replicate is repeatably measured at each time point. In general an equal number of time measurements and technical replicates per biological replicate are not required.

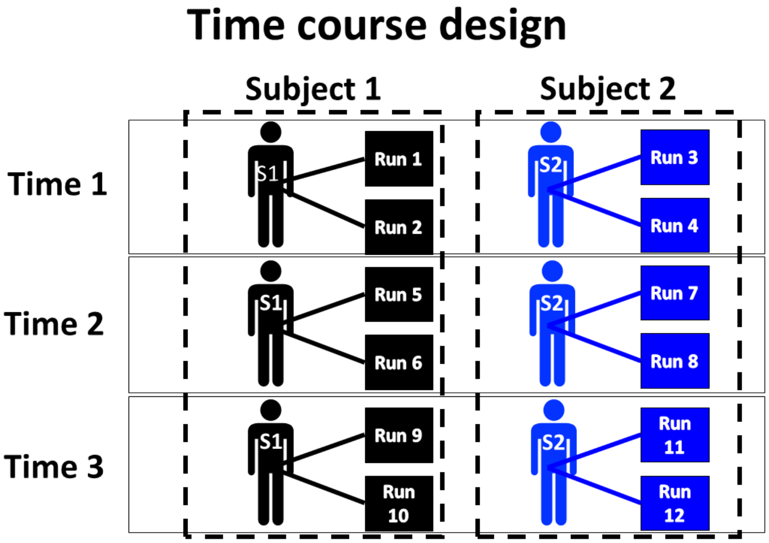

Supplementary Fig. 6: **Example time course design** 12 total MS runs spread across 3 *Time* points and 2 biological replicates. Both biological replicates are measured using two technical replicates at each *Time*.

1.3.2 Annotation file

When creating an annotation file for time course designs, the user must make sure that the same biological replicate id is repeated for each time point it was measured at. **Supplementary Table 2** shows an example annotation file for the experimental design in **Supplementary Fig. 6**.

1.3.3 Statistical details

Supplementary Fig. 7 shows the corresponding linear mixed effects model for an experiment with both biological and technical replicates. **Supplementary Fig. 8** shows the corresponding ANOVA decomposition in balanced designs. Pairwise comparisons of conditions based on the decomposition in **Supplementary Fig. 8** is shown in **Supplementary Fig. 9**

The whole plot model is

$$z_{ijk} = \mu + Condition_i + Subject_j + Condition_i \times Subject_j + \psi_{ijk}, \text{where} \tag{1}$$

Condition	BioReplicate	Run
Time 1	S1	Run 1
Time 1	S1	Run 2
Time 1	S2	Run 3
Time 1	S2	Run 4
Time 2	S1	Run 5
Time 2	S1	Run 6
Time 2	S2	Run 7
Time 2	S2	Run 8
Time 3	S1	Run 9
Time 3	S1	Run 10
Time 3	S2	Run 11
Time 3	S2	Run 12

Supplementary Table 2: **Example annotation file for the time course design in Supplementary Fig. 6**. Note that *Time* is entered into the "Condition" column. The ids in the "BioReplicate" column are repeated for every *Time* point they were measured. This is in contrast to a group comparison design where each biological replicate was only entered used for a single condition.

$$y_{ijkl} = \overbrace{\mu + \mathbf{Condition_i} + \underbrace{\mathbf{Subject_j} + \mathbf{Condition} \times \mathbf{Subject_{ij}}}_{\text{Whole-plot biological variation}} + \underbrace{\mathbf{Run_{ijk}}}_{\text{Whole-plot technical variation}}}^{\text{Whole plot}} + \overbrace{\mathbf{Feature_l} + \underbrace{\mathbf{Run} \times \mathbf{Feature_{ijkl}}}_{\text{Subplot error}}}^{\text{Subplot}}$$

where

$$\textstyle\sum_{i=1}^{I} \mathbf{Condition}_i = 0,\ \mathbf{Subject_j} \overset{\mathbf{iid}}{\sim} \mathcal{N}(\mathbf{0}, \sigma^2_{\mathbf{Subject}}),\ \mathbf{Condition} \times \mathbf{Subject_{ij}} \overset{iid}{\sim} \mathcal{N}(0, \sigma^2_{Condition \times Subject}),$$
$$\mathbf{Run_{ijk}} \overset{\mathbf{iid}}{\sim} \mathcal{N}(\mathbf{0}, \sigma^2_{\mathbf{Run}}),\ \textstyle\sum_{j=1}^{L} \mathbf{Feature}_l = 0,\ \mathbf{Run} \times \mathbf{Feature_{ijkl}} = \epsilon_{\mathbf{ijkl}} \overset{\mathbf{iid}}{\sim} \mathcal{N}(\mathbf{0}, \sigma^2_{\epsilon})$$

Supplementary Fig. 7: **Full linear mixed-effects model for one protein in Supplementary Fig. 11.** y_{ijklm} is the $\log_2$-intensity of the ith *Condition*, jth *Subject*, kth *Run*, lth *Feature*. μ is the mean $\log_2$-intensity of the protein. The model decomposes the variation in the $\log_2$-intensities into contributions from each source. Pink: sources of variation in the whole plot. Yellow: sources of variation in the subplot.

$$\textstyle\sum_{i=1}^{I} Condition_i = 0,\ Subject_j \overset{iid}{\sim} \mathcal{N}(0, \sigma^2_{Subject}),\ Condition \times Subject_{ij} \overset{iid}{\sim} \mathcal{N}(0, \sigma^2_{Condition \times Subject}),\ \psi_{ijk} \overset{iid}{\sim} \mathcal{N}(0, \sigma^2_{\psi})$$

If the experiment has one technical replicate, the model is substituted with

$$z_{ij} = \mu + Condition_i + Subject_j + \psi_{ij}, \text{where} \tag{2}$$
$$\sum_{i=1}^{I} Condition_i = 0,\ Subject_j \overset{iid}{\sim} \mathcal{N}(0, \sigma^2_{Subject}), \psi_{ij} \overset{iid}{\sim} \mathcal{N}(0, \sigma^2_{\psi})$$

Model term	Sum of squares (SS)	Degrees of freedom	Expected mean squares
Condition	$JKL_i(\bar{y}_{i...} - \bar{y}_{....})^2$	$I-1$	σ^2_ϵ + Lσ^2_{Run} + KL$\sigma^2_{Condition \times Subject}$ + $\frac{JKL \sum Condition^2_i}{I-1}$
Subject	$IKL \sum_j (\bar{y}_{.j..} - \bar{y}_{....})^2$	$J-1$	σ^2_ϵ + Lσ^2_{Run} + IKL$\sigma^2_{Subject}$
Condition × Subject	$KL \sum_{ij} (\bar{y}_{ij..} - \bar{y}_{i...} - \bar{y}_{.j..} + \bar{y}_{...})^2$	$(I-1)(J-1)$	σ^2_ϵ + Lσ^2_{Run} + KL$\sigma^2_{Condition \times Subject}$
Wholeplot error (Run)	$L \sum_{ijk} (\bar{y}_{ijk.} - \bar{y}_{ij..})^2$	$IJ(K-1)$	σ^2_ϵ + Lσ^2_{Run}
Feature	$IKJ \sum_l (\bar{y}_{...l} - \bar{y}_{....})^2$	$L-1$	σ^2_ϵ + $\frac{IJK \sum Feature_l{}^2}{L-1}$
Subplot error	$\sum_{ijkl} (y_{ijkl} - \bar{y}_{ijk.} - \bar{y}_{...l} + \bar{y}_{....})^2$	$(IJK-1)(L-1)$	σ^2_ϵ
Total	SS_{Total}	$IJKL-1$	

Supplementary Fig. 8: **Analysis of variance for the the model in Supplementary Fig. 7, in the special case of balanced designs.** Horizontal line separates whole plot and subplot, which have a different structure of random error. Gray box indicates the error term relevant when comparing conditions.

Pairwise comparison	Estimated log-fold change	Theoretical variance	Estimated variance	Degrees of freedom
Condition$_i$ − Condition$_{i'}$	$\bar{y}_{i...} - \bar{y}_{i'...}$	$\frac{2\sigma^2_\epsilon}{JKL} + \frac{2\sigma^2_{Run}}{JK} + \frac{2\sigma^2_{Condition \times Subject}}{J}$	$\frac{2}{JKL} \times \frac{SS_{Condition \times Subject}}{(I-1)(J-1)}$	$(I-1)(J-1)$

Supplementary Fig. 9: **ANOVA-based pairwise comparison of conditions, based on Supplementary Fig. 8.** SS indicates sum of squares.

1.4 Extension to label-free paired designs

1.4.1 Experimental design

In label-free experiments with paired designs, the subplot structure is the same as in a group comparison and time series designs. A paired design is very similar to a time series design, where the same biological replicate is measured in multiple conditions s (such as healthy biopsy and disease biopsy). However, it differs in that there is generally no notion of time specifically encoded into a paired design. The whole plot for a paired design is the same as the time series. It contains repeated measurements the same biological replicate, across *Conditions*. **Supplementary Fig. 10** shows an example paired design experiment with two biological replicates and two conditions.

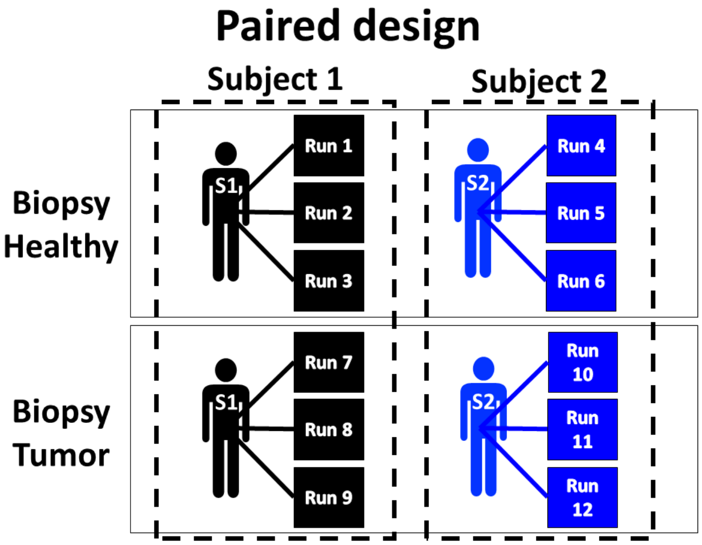

Supplementary Fig. 10: **Example paired experimental design.** 2 biological replicates were measured at 2 conditions, healthy biopsy and tumor biopsy. There were 3 technical replicates per biological replicate at each condition, resulting in 12 total mass spectrometry runs.

1.4.2 Annotation file

The annotation file for a paired designs is very similar to a time course file. The user must make sure that the same biological replicate id is repeated for each condition it was measured at. **Supplementary Fig. 10** shows an example annotation file for the experimental design in **Supplementary Fig. 10**.

1.4.3 Statistical details

The statistical details of paired designs is the same as those for time series designs because they are both designed around repeated measures. Thus see **Supplementary Sec. 1.3.3** for statistical details.

**Condition**	**BioReplicate**	**Run**
Biopsy Healthy	S1	Run 1
Biopsy Healthy	S1	Run 2
Biopsy Healthy	S1	Run 3
Biopsy Healthy	S2	Run 4
Biopsy Healthy	S2	Run 5
Biopsy Healthy	S2	Run 6
Biopsy Tumor	S1	Run 7
Biopsy Tumor	S1	Run 8
Biopsy Tumor	S1	Run 9
Biopsy Tumor	S2	Run 10
Biopsy Tumor	S2	Run 11
Biopsy Tumor	S2	Run 12

Supplementary Table 3: **Example annotation file for the paired design in Supplementary Fig. 10**.

1.5 Extension to group comparison experiment with labeled reference peptides

1.5.1 Experimental design

The use of labeling changes the subplot structure of the experiment as compared to label-free experiments, however the whole plot remains the same. **Supplementary Fig. 11** shows a representative data structure for a group comparison experiment with labeled reference peptides. It has additional L rows for labeled reference counterparts of each endogenous feature. $m = \{0, 1\}$ is the index of *Label*, where 0 denotes labeled reference features and 1 denotes endogenous features.

Note that we do not include a separate experimental design figure here because the setup will be the same as in **Supplementary Fig. 2**.

1.5.2 Annotation file

Group comparison experiments with labeled reference peptides are different from the other experimental designs detailed previously in that they do not require a unique annotation file. Please use the annotation design listed in **Supplementary Sec. 1.2.2** for group comparison designs.

1.5.3 Statistical details

Supplementary Fig. 12 details the corresponding linear mixed-effects model.

Whole plot

Subplot

		Condition$_1$										· · ·	Condition$_I$									
		Subject$_1$			Subject$_2$			· · ·	Subject$_J$			· · ·	Subject$_{(I-1)J+1}$			Subject$_{(I-1)J+2}$			· · ·	Subject$_{IJ}$		
		Run$_1$	Run$_2$	Run$_3$	Run$_4$	Run$_5$	Run$_6$	· · ·	Run$_{JK-2}$	Run$_{JK-1}$	Run$_{JK}$	· · ·	Run$_{(I-1)JK+1}$	Run$_{(I-1)JK+2}$	Run$_{(I-1)JK+3}$	Run$_{(I-1)JK+4}$	Run$_{(I-1)JK+5}$	Run$_{(I-1)JK+6}$	· · ·	Run$_{IJK-2}$	Run$_{IJK-1}$	Run$_{IJK}$
Endogenous	Feature$_1$	y	y	y	y	y	y	· · ·	y	y	y	· · ·	y	y	y	y	y	y	· · ·	y	y	y
	Feature$_2$	y	y	y	y	y	**NA**	· · ·	y	y	y	· · ·	y	y	y	y	y	y	· · ·	y	y	y
	· · ·	· · ·	· · ·	· · ·	· · ·	· · ·	· · ·	· · ·	· · ·	· · ·	· · ·	· · ·	· · ·	· · ·	· · ·	· · ·	· · ·	· · ·	· · ·	· · ·	· · ·	· · ·
	Feature$_L$	y	**Cens**	y	**Cens**	**Cens**	y	· · ·	**Cens**	y	y	· · ·	**Cens**	y	y	y	y	y	· · ·	y	**NA**	y
Reference	Feature$_1$	y	y	y	y	y	y	· · ·	y	y	y	· · ·	y	y	y	y	y	y	· · ·	y	y	y
	Feature$_2$	y	y	y	y	y	y	· · ·	y	y	y	· · ·	y	y	y	y	**NA**	y	· · ·	y	y	y
	· · ·	· · ·	· · ·	· · ·	· · ·	· · ·	· · ·	· · ·	· · ·	· · ·	· · ·	· · ·	· · ·	· · ·	· · ·	· · ·	· · ·	· · ·	· · ·	· · ·	· · ·	· · ·
	Feature$_L$	y	y	**NA**	y	y	y	· · ·	y	y	y	· · ·	y	y	y	y	y	y	· · ·	y	y	y

Supplementary Fig. 11: **Data structure for one protein, from an experiment with labeled reference peptides with a group comparison design and technical replicates.** y is the $\log_2$-intensity of the observed feature in each cell. "Cens" are missing values censored for reasons of low abundance, and "NA" are values missing at random. Pink represents the biological variation (whole plot); yellow represents the technological variation (subplot).

$$\overbrace{y_{ijklm} = \mu + \textbf{Condition}_i + \underbrace{\textbf{Subject(Condition)}_{\mathbf{j(i)}}}_{\substack{\text{Whole-plot}\\\text{biological}\\\text{variation}}} + \underbrace{\textbf{Run}_{\mathbf{ijk}}}_{\substack{\text{Whole-plot}\\\text{technical}\\\text{variation}}}}^{\text{Whole plot}} + \overbrace{\textbf{Label}_{\mathbf{m}} + \textbf{Run} \times \textbf{Label}_{ijkm} + \textbf{Feature}_{\mathbf{l}} + \underbrace{\epsilon_{ijklm}}_{\substack{\text{Subplot}\\\text{error}}}}^{\text{Subplot}}$$

where

$$\sum_{i=1}^{I} \textbf{Condition}_i = 0,\ \textbf{Subject(Condition)}_{\mathbf{j(i)}} \overset{\text{iid}}{\sim} \mathcal{N}(\mathbf{0}, \sigma^{\mathbf{2}}_{\textbf{Subject}}),\ \textbf{Run}_{\mathbf{ijk}} \overset{\text{iid}}{\sim} \mathcal{N}(\mathbf{0}, \sigma^{\mathbf{2}}_{\textbf{Run}}),$$

$$\sum_{j=1}^{L} \textbf{Feature}_l = 0,\ \sum_{m=0}^{1} \textbf{Label}_m = 0,\ \textbf{Run} \times \textbf{Feature}_{\mathbf{ijkl}} = \epsilon_{\mathbf{ijkl}} \overset{\text{iid}}{\sim} \mathcal{N}(\mathbf{0}, \sigma^{\mathbf{2}}_{\epsilon})$$

Supplementary Fig. 12: **Full linear mixed-effects model for one protein in Supplementary Fig. 11.** y_{ijklm} is the $\log_2$-intensity of the ith *Condition*, jth *Subject*, kth *Run*, lth *Feature*, and mth *Label*. μ is the mean $\log_2$-intensity of the protein. The model decomposes the variation in the $\log_2$-intensities into contributions from each source. Pink: sources of variation in the whole plot. Yellow: sources of variation in the subplot.

The model for subplot summarization in Step 1 of **Figure 1**(c) is replaced with

$$y_{ijklm} = \mu + Run_{ijk} + Label_m + Run \times Label_{ijkm} + Feature_l + error_{ijklm}, \tag{3}$$

where y_{ijkl} include imputed $\log_2$-intensities, μ is the median $\log_2$-intensity across features and runs, the medians of Run_{ijk}, $Label_m$, $Run \times Label_{ijkm}$, $Feature_l$ and $error_{ijkl}$ are 0, and the errors are independent. The $Run \times Label_{ijkm}$ interaction expresses differences in quantitative profiles of features with different labels. Note that censoring of peak intensities is only applied to endogenous peaks, and missing peak intensities from reference peaks are assumed to be missing at random.

Parameters in Eq. (3) are estimated using Tukey Median Polish. The summarized intensities per label and run are

$$\begin{aligned} \hat{y}_{ijk.1} &= \widehat{Label}_1 + \widehat{Run}_{ijk} + \widehat{Run \times Label}_{ijk1} \text{ for endogenous intensities} \\ \hat{y}_{ijk.0} &= \widehat{Label}_0 + \widehat{Run}_{ijk} + \widehat{Run \times Label}_{ijk0} \text{ for reference intensities} \end{aligned} \tag{4}$$

Next, summarized endogenous $\log_2$-intensities are adjusted with respect to their reference counterparts, using the following linear combination of the estimates

$$z_{ijk} = \hat{y}_{ijk.1} - \Big(\hat{y}_{ijk.0} - median_{ijk.0}(\widehat{Label}_0 + \widehat{Run}_{ijk} + \widehat{Run \times Label}_{ijk0})\Big) \tag{5}$$

The adjustment equalizes the median of summarized reference $\log_2$-intensities across runs, and shifts the summarized $\log_2$-intensities of the endogenous features in the same run by the same amount. In rare situations when all the reference intensities of a protein are missing in a run, the endogenous intensities are left unadjusted.

Supplementary Note 2 : MSstats v2.0 versus MSstats v4.0

This section contrasts model-based inference in MSstats v2.0 [3, 4, 5] and MSstats v4.0 (this manuscript). We consider four families of models: v2.0 fixed effects, v2.0 mixed effects, full model (**Figure 1**(b)) and v4.0 whole plot model (advocated in this manuscript, **Figure 1**(c)). We distinguish experimental designs with group comparison and paired/time course, with and without biological and technical replicates, label-free and using labeled reference peptides.

This section aims to contrast the models, and the model-based inference. Therefore, imputation and robust summarization implemented in v4.0 are omitted from this discussion. Parameters of all the models are estimated with the same ANOVA and likelihood-based procedures.

2.1 Differences in theoretical ANOVA-based inference in balanced designs

Summary: MSstats v2.0 with reduced scope of biological replication overfits. Theoretical inference from MSstats v2.0 with expanded scope of biological replication coincides with MSstats v4.0 in group comparison and paired/time course designs, but cannot be used with other designs.

The following pages detail the models in MSstats v2.0 and v4.0 for various experimental designs. The models differ in reduced (v2.0 fixed) versus expanded (v2.0 mixed, v4.0) scope of biological replication. Reduced scope of biological replication restricts the conclusions to the subjects in the study (as opposed to the larger underlying population). This is useful in earlier days of technology development, when experiments have few biological replicates and noisy measurements. However, reduced scope of biological replication under-represents the full extent of variation in the population, and limits the between-study reproducibility of the results [6]. Retiring this option is one of the reasons for introducing v4.0.

The models differ in presence of **Feature** $\times$ **Condition** statistical interaction (v2.0) and presence of **Run** (v4.0). The **Feature** $\times$ **Condition** term in v2.0 was necessary in earlier days, when features were noisy due to both technological limitations (e.g., interferences) and suboptimal computational procedures (e.g, difficulties in identifying and quantifying peaks). Recent advances reduced these artifacts, to the point that the interaction term became unnecessary. The term **Run** is proposed in v4.0 to reflect the split-plot nature of the experimental design.

The following pages detail ANOVA-based inference for pairwise comparisons of conditions. As can be seen from **Supplementary Fig. 13**, reduced scope of biological replication in v2.0 under-estimates the population variance (i.e., it conflates the biological and the technological variation, and only estimates one variance component), and over-estimates the degrees of freedom.

In label-free experiments with group comparison or paired/time course designs, ANOVA-based inference in v2.0 with expanded scope of biological replication coincides with inference in v4.0 (**Supplementary Fig. 13-14**). However, the models in v2.0 cannot be accurately extended to other designs. In particular, in experiments with controlled mixtures without biological replicates, all the models in v2.0 conflate the within-run and between-run variation, under-estimate the variance and over-estimate the degrees of freedom (**Supplementary Fig. 15**). Similarly, the models in v2.0 are not applicable to experiments with labeled reference peptides. In contrast, inference from the models in v4.0 appropriately distinguishes different sources of variation. Moreover, ANOVA-based inference from the full model and the whole plot model coincides for all the designs. This observation is the basis for advocating for the whole plot model in this manuscript.

Balanced label-free group comparison design with biological and technical replicates

(a) Linear FIXED effects model in MSstats v2.0 :

$$y_{ijkl} = \mu + \textbf{Condition}_i + \textbf{Subject(Condition)}_{j(i)} + \textbf{Feature}_l + (\textbf{Feature} \times \textbf{Condition})_{ijl} + \epsilon_{ijkl}$$
$$\text{where } \sum_{i=1}^{I} \textbf{Condition}_i = 0,\ \sum_{j=1}^{J} \textbf{Subject}_j = 0,\ \sum_{l=1}^{L} \textbf{Feature}_l = 0,$$
$$\sum_{j=1}^{J} \textbf{Feature} \times \textbf{Condition}_{ijl} = 0,\ \sum_{i=1}^{I} \textbf{Feature} \times \textbf{Condition}_{ijl} = 0,$$
$$\epsilon_{ijkl} \overset{iid}{\sim} \mathcal{N}(0, \sigma_\epsilon^2)$$

(b) Linear MIXED effects model in MSstats v2.0 :

$$y_{ijkl} = \mu + \textbf{Condition}_i + \textbf{Subject(Condition)}_{j(i)} + \textbf{Feature}_l + (\textbf{Feature} \times \textbf{Condition})_{ijl} + \epsilon_{ijkl}$$
$$\text{where } \sum_{i=1}^{I} \textbf{Condition}_i = 0,\ \sum_{l=1}^{L} \textbf{Feature}_l = 0,$$
$$\sum_{j=1}^{J} \textbf{Feature} \times \textbf{Condition}_{il} = 0,\ \sum_{i=1}^{I} \textbf{Feature} \times \textbf{Condition}_{il} = 0$$
$$\textbf{Subject(Condition)}_{j(i)} \overset{iid}{\sim} \mathcal{N}(0, \sigma_{S2}^2),\ \epsilon_{ijkl} \overset{iid}{\sim} \mathcal{N}(0, \sigma_2^2)$$

(c) FULL model in MSstats v4.0 :

$$y_{ijkl} = \mu + \textbf{Condition}_i + \textbf{Subject(Condition)}_{j(i)} + \textbf{Run}_{ijk} + \textbf{Feature}_l + \epsilon_{ijkl}$$
$$\text{where } \sum_{i=1}^{I} \textbf{Condition}_i = 0,\ \sum_{l=1}^{L} \textbf{Feature}_l = 0,$$
$$\textbf{Subject(Condition)}_{j(i)} \overset{iid}{\sim} \mathcal{N}(0, \sigma_{S3}^2),\ \textbf{Run}_{ijk} \overset{iid}{\sim} \mathcal{N}(0, \sigma_\omega^2),\ \epsilon_{ijkl} \overset{iid}{\sim} \mathcal{N}(0, \sigma_3^2)$$

(d) Whole plot model in MSstats v4.0

$$z_{ijk} = \mu + \textbf{Condition}_i + \textbf{Subject(Condition)}_{j(i)} + \xi_{ijk}$$
$$\text{where } \sum_{i=1}^{I} \textbf{Condition}_i = 0,$$
$$\textbf{Subject(Condition)}_{j(i)} \overset{iid}{\sim} \mathcal{N}(0, \sigma_\tau^2),\ \xi_{ijk} \overset{iid}{\sim} \mathcal{N}(0, \sigma_\xi^2)$$

$Condition_i - Condition_{i'}$	Estimated log-fold change	Theoretical variance	Estimated variance	Degrees of freedom
(b) Linear FIXED model v2.0	$\bar{y}_{i\ldots} - \bar{y}_{i'\ldots}$	$\frac{2\sigma_\epsilon^2}{JKL}$	$\frac{2}{JKL} \times \frac{\sum\sum\sum\sum_{ijkl}(\bar{y}_{ijkl} - \bar{y}_{ij..} - \bar{y}_{i..l} + \bar{y}_{i...})^2}{IJKL - IL - IJ + I}$	$IJKL - IL - IJ + I$
(b) Linear MIXED model v2.0	$\bar{y}_{i\ldots} - \bar{y}_{i'\ldots}$	$\frac{2\sigma_2^2}{JKL} + \frac{2\sigma_{S2}^2}{J}$	$\frac{2}{JKL} \times \frac{KL\sum\sum_{ij}(\bar{y}_{ij..} - \bar{y}_{i...})^2}{I(J-1)}$	$I(J-1)$
(c) FULL model v4.0	$\bar{y}_{i\ldots} - \bar{y}_{i'\ldots}$	$\frac{2\sigma_3^2}{JKL} + \frac{2\sigma_\omega^2}{JK} + \frac{2\sigma_{S3}^2}{J}$	$\frac{2}{JKL} \times \frac{KL\sum\sum_{ij}(\bar{y}_{ij..} - \bar{y}_{i...})^2}{I(J-1)}$	$I(J-1)$
(d) Whole plot model v4.0	$\bar{z}_{i..} - \bar{z}_{i'..}$	$\frac{2\sigma_\xi^2}{JK} + \frac{2\sigma_\tau^2}{J}$	$\frac{2}{JK} \times \frac{K\sum\sum_{ij}(\bar{z}_{ij.} - \bar{z}_{i..})^2}{I(J-1)}$	$I(J-1)$

Supplementary Fig. 13: **ANOVA-based inference for pairwise comparisons in balanced label-free designs** The model in (a) under-estimates the variation in the population and over-estimates the degrees of freedom. Theoretical inference from models in (b), (c) and (d) coincide in balanced designs.

Note: In experiments with only one technical replicate, models in (a) and (b) are unchanged. In (c), the term $\textbf{Run}_{ijk}$ and its variance component are not estimable, and are eliminated. In (d), the term $\textbf{Subject(Condition)}_{j(i)}$ and its variance component are not estimable and are eliminated. As the result, the errors in (c) and (d) combine the biological and the between-run variation. Comparisons in **Supplementary Fig. 13** and unchanged, with $K = 1$.

Balanced label-free time course or paired design with biological and technical replicates

(a) Linear FIXED effects model in MSstats v2.0 :

$$
\begin{aligned}
y_{ijkl} \ &= \mu + \textbf{Condition}_i + \textbf{Subject}_\mathbf{j} + \textbf{Condition} \times \textbf{Subject}_\mathbf{ij} + \textbf{Feature}_l + (\textbf{Feature} \times \textbf{Condition})_{ijl} + \epsilon_{ijkl} \\
&\text{where } \ \textstyle\sum_{i=1}^{I} \textbf{Condition}_i = 0, \ \sum_{j=1}^{J} \textbf{Subject}_j = 0, \\
&\textstyle\sum_{i=1}^{I} \textbf{Condition} \times \textbf{Subject}_{ij} = 0, \ \sum_{j=1}^{J} \textbf{Condition} \times \textbf{Subject}_{ij} = 0, \\
&\textstyle\sum_{l=1}^{L} \textbf{Feature}_l = 0, \ \sum_{j=1}^{J} \textbf{Feature} \times \textbf{Condition}_{ijl} = 0, \ \sum_{i=1}^{I} \textbf{Feature} \times \textbf{Condition}_{ijl} = 0 \\
&\epsilon_{ijkl} \overset{iid}{\sim} \mathcal{N}(0, \sigma_\epsilon^2)
\end{aligned}
$$

(b) Linear MIXED effects model in MSstats v2.0 :

$$
\begin{aligned}
y_{ijkl} \ &= \mu + \textbf{Condition}_i + \textbf{Subject}_\mathbf{j} + \textbf{Condition} \times \textbf{Subject}_\mathbf{ij} + \textbf{Feature}_l + (\textbf{Feature} \times \textbf{Condition})_{ijl} + \epsilon_{ijkl} \\
&\text{where } \ \textstyle\sum_{i=1}^{I} \textbf{Condition}_i = 0, \ \sum_{l=1}^{L} \textbf{Feature}_l = 0, \\
&\textstyle\sum_{j=1}^{J} \textbf{Feature} \times \textbf{Condition}_{ijl} = 0, \ \sum_{i=1}^{I} \textbf{Feature} \times \textbf{Condition}_{ijl} = 0 \\
&\textbf{Subject}_j \overset{iid}{\sim} \mathcal{N}(0, \sigma_{S2}^2), \ \textbf{Condition} \times \textbf{Subject}_{ij} \overset{iid}{\sim} \mathcal{N}(0, \sigma_{CS2}^2), \ \epsilon_{ijkl} \overset{iid}{\sim} \mathcal{N}(0, \sigma_2^2)
\end{aligned}
$$

(c) FULL model in MSstats v4.0 :

$$
\begin{aligned}
y_{ijkl} \ &= \mu + \textbf{Condition}_i + \textbf{Subject}_\mathbf{j} + \textbf{Condition} \times \textbf{Subject}_\mathbf{ij} + \textbf{Run}_{ijk} + \textbf{Feature}_l + \epsilon_{ijl} \\
&\text{where } \ \textstyle\sum_{i=1}^{I} \textbf{Condition}_i = 0, \ \sum_{l=1}^{L} \textbf{Feature}_l = 0, \\
&\textbf{Subject}_j \overset{iid}{\sim} \mathcal{N}(0, \sigma_{S3}^2), \ \textbf{Condition} \times \textbf{Subject}_{ij} \overset{iid}{\sim} \mathcal{N}(0, \sigma_{CS3}^2), \ \textbf{Run}_{ijk} \overset{iid}{\sim} \mathcal{N}(0, \sigma_\omega^2), \ \epsilon_{ijkl} \overset{iid}{\sim} \mathcal{N}(0, \sigma_3^2)
\end{aligned}
$$

(d) Whole plot model in MSstats v4.0 :

$$
\begin{aligned}
z_{ijk} \ &= \mu + \textbf{Condition}_i + \textbf{Subject}_j + \textbf{Condition} \times \textbf{Subject}_\mathbf{ij} + \xi_{ijk} \\
&\text{where } \ \textstyle\sum_{i=1}^{I} \textbf{Condition}_i = 0, \\
&\textbf{Subject}_j \overset{iid}{\sim} \mathcal{N}(0, \sigma_\tau^2), \ \textbf{Condition} \times \textbf{Subject}_{ij} \overset{iid}{\sim} \mathcal{N}(0, \sigma_\phi^2), \ \xi_{ijk} \overset{iid}{\sim} \mathcal{N}(0, \sigma_\xi^2)
\end{aligned}
$$

$Condition_i - Condition_{i'}$	Estimated log-fold change	Theoretical variance	Estimated variance	Degrees of freedom
(a) Linear FIXED model v2.0	$\bar{y}_{i\ldots} - \bar{y}_{i'\ldots}$	$\frac{2\sigma_\epsilon^2}{JKL}$	$\frac{2}{JKL} \times \frac{\sum\sum\sum\sum_{ijkl}(\bar{y}_{ijkl} - \bar{y}_{ij..} - \bar{y}_{i..l} + \bar{y}_{i\ldots})^2}{IJKL-IL-IJ+I}$	$IJKL - IL - IJ + I$
(b) Linear MIXED model v2.0	$\bar{y}_{i\ldots} - \bar{y}_{i'\ldots}$	$\frac{2\sigma_2^2}{JKL} + \frac{2\sigma_{CS2}^2}{J}$	$\frac{2}{JKL} \times \frac{KL\sum\sum_{ij}(\bar{y}_{ij..} - \bar{y}_{i\ldots} - \bar{y}_{.j..} + \bar{y}_{\ldots.})^2}{(I-1)(J-1)}$	$(I-1)(J-1)$
(c) FULL model v4.0	$\bar{y}_{i\ldots} - \bar{y}_{i'\ldots}$	$\frac{2\sigma_3^2}{JKL} + \frac{2\sigma_\omega^2}{JK} + \frac{2\sigma_{CS2}^2}{J}$	$\frac{2}{JKL} \times \frac{KL\sum\sum_{ij}(\bar{y}_{ij..} - \bar{y}_{i\ldots} - \bar{y}_{.j..} + \bar{y}_{\ldots})^2}{(I-1)(J-1)}$	$(I-1)(J-1)$
(d) Whole plot model v4.0	$\bar{z}_{i..} - \bar{z}_{i'..}$	$\frac{2\sigma_\xi^2}{JK} + \frac{2\sigma_\phi^2}{J}$	$\frac{2}{JK} \times \frac{K\sum\sum_{ij}(\bar{z}_{ij.} - \bar{z}_{i..} - \bar{z}_{.j.} + \bar{z}_{\ldots})^2}{(I-1)(J-1)}$	$(I-1)(J-1)$

Supplementary Fig. 14: **ANOVA-based inference for pairwise comparisons in balanced label-free designs** The model in (a) under-estimates the variation in the population and over-estimates the degrees of freedom. Theoretical inference from models in (b), (c) and (d) coincide in balanced designs.

Note: In experiments with only one technical replicate, models in (a) and (b) are unchanged. In (c), the term $\textbf{Run}_{ijk}$ and its variance component are not estimable, and are eliminated. In (d), the term $\textbf{Condition} \times \textbf{Subject}_\mathbf{ij}$ and its variance component are not estimable and are eliminated. As the result, the errors in (c) and (d) combine the biological and the between-run variation. Comparisons in **Supplementary Fig. 14** and unchanged, with $K = 1$.

Balanced label-free group comparison design with technical replicates only (controlled mixtures)

(a) Linear FIXED effects model and linear MIXED effects model coincide in MSstats v2.0 :

$$y_{ikl} = \mu + \textbf{Condition}_i + \textbf{Feature}_l + (\textbf{Feature} \times \textbf{Condition})_{ikl} + \epsilon_{ikl}$$

where $\sum_{i=1}^{I} \textbf{Condition}_i = 0$, $\sum_{l=1}^{L} \textbf{Feature}_l = 0$,

$\sum_{i=1}^{I} \textbf{Feature} \times \textbf{Condition}_{ikl} = 0$, $\sum_{k=1}^{K} \textbf{Feature} \times \textbf{Condition}_{ikl} = 0$

$\epsilon_{ikl} \overset{iid}{\sim} \mathcal{N}(0, \sigma_2^2)$

(b) FULL model in MSstats v4.0 :

$$y_{ikl} = \mu + \textbf{Condition}_i + \textbf{Run}_{ik} + \textbf{Feature}_l + \epsilon_{ikl}$$

where $\sum_{i=1}^{I} \textbf{Condition}_i = 0$, $\sum_{l=1}^{L} \textbf{Feature}_l = 0$,

$\textbf{Run}_{ik} \overset{iid}{\sim} \mathcal{N}(0, \sigma_\omega^2)$, $\epsilon_{ikl} \overset{iid}{\sim} \mathcal{N}(0, \sigma_3^2)$

(c) Whole plot model in MSstats v4.0 :

$$z_{ik} = \mu + \textbf{Condition}_i + \xi_{ik}$$

where $\sum_{i=1}^{I} \textbf{Condition}_i = 0$,

$\xi_{ik} \overset{iid}{\sim} \mathcal{N}(0, \sigma_\xi^2)$

$Condition_i - Condition_{i'}$	Estimated log-fold change	Theoretical variance	Estimated variance	Degrees of freedom
(a) Linear FIXED/MIXED v2.0	$\bar{y}_{i..} - \bar{y}_{i'..}$	$\frac{2\sigma_2^2}{KL}$	$\frac{2}{KL} \times \frac{\sum\sum\sum_{ikl}(y_{ikl} - \bar{y}_{i.l})^2}{IL(K-1)}$	$IL(K-1)$
(b) FULL model v4.0	$\bar{y}_{i..} - \bar{y}_{i'..}$	$\frac{2\sigma_3^2}{KL} + \frac{2\sigma_\omega^2}{K}$	$\frac{2}{KL} \times \frac{L\sum\sum_{ik}(\bar{y}_{ik.} - \bar{y}_{i..})^2}{I(K-1)}$	$I(K-1)$
(c) Whole plot model v4.0	$\bar{z}_{i.} - \bar{z}_{i'.}$	$\frac{2\sigma_\xi^2}{K}$	$\frac{2}{K} \times \frac{\sum\sum_{ik}(z_{ik} - \bar{z}_{i.})^2}{I(K-1)}$	$I(K-1)$

Supplementary Fig. 15: **ANOVA-based inference for pairwise comparisons in balanced label-free designs** The model in (a) under-estimates the variation in the population and over-estimates the degrees of freedom. Theoretical inference from models in (b) and (c) coincide in balanced designs.

References

[1] A. L. Oberg and O. Vitek. "Statistical design of quantitative mass spectrometry-based proteomic experiments". In: *Journal of Proteome Research* 8 (2009), p. 2144.

[2] D. C. Montgomery. *Design and Analysis of Experiments*. 8th. New Jersey, USA: John Wiley & Sons, Inc., 2013.

[3] M. Choi et al. "MSstats: an R package for statistical analysis of quantitative mass spectrometry-based proteomic experiments". In: *Bioinformatics* 30.17 (2014), pp. 2524–2526.

[4] T. Clough et al. "Statistical protein quantification and significance analysis in label-free LC-MS experiments with complex designs". In: *BMC Bioinformatics* 13 (2012), S6.

[5] C.- Y. Chang et al. "Protein significance analysis in selected reaction monitoring (SRM) measurements". In: *Molecular & Cellular Proteomics* 11.4 (Apr. 2012), p. M111.014662.

[6] T. Yarkoni. "The generalizability crisis". In: *Behavioral and Brain Sciences* 45 (2022), e1.
